# Diversity and biological activity of culturable endophytic bacteria associated with marigold (*Calendula officinalis* L.)

**DOI:** 10.3934/microbiol.2021021

**Published:** 2021-09-13

**Authors:** Vyacheslav Shurigin, Burak Alaylar, Kakhramon Davranov, Stephan Wirth, Sonoko Dorothea Bellingrath-Kimura, Dilfuza Egamberdieva

**Affiliations:** 1 Department of Microbiology and Biotechnology, Faculty of Biology, National University of Uzbekistan, 100174, Tashkent, Uzbekistan; 2 Department of Molecular Biology and Genetics, Faculty of Arts and Sciences, Agri Ibrahim Cecen University, 04100, Agri, Turkey; 3 Institute of Microbiology of the Academy of Sciences of the Republic of Uzbekistan, 100128 Tashkent, Uzbekistan; 4 Leibniz Centre for Agricultural Landscape Research (ZALF), 15374 Müncheberg, Germany; 5 Faculty of Life Science, Humboldt University of Berlin, 14195, Berlin, Germany

**Keywords:** antagonism, auxin, endophyte, marigold, plant growth promotion

## Abstract

Endophytes colonizing plant tissue play an essential role in plant growth, development, stress tolerance and plant protection from soil-borne diseases. In this study, we report the diversity of cultivable endophytic bacteria associated with marigold (*Calendula officinalis* L.) by using 16S rRNA gene analysis and their plant beneficial properties. A total of 42 bacterial isolates were obtained from plant tissues of marigold. They belonged to the genera *Pantoea, Enterobacter, Pseudomonas, Achromobacter, Xanthomonas, Rathayibacter, Agrobacterium, Pseudoxanthomonas*, and *Beijerinckia*. Among the bacterial strains, *P. kilonensis* FRT12, and *P. rhizosphaerae* FST5 showed moderate or vigorous inhibition against three tested plant pathogenic fungi, *F. culmorum, F. solani* and *R. solani*. They also demonstrated the capability to produce hydrolytic enzymes and indole-3-acetic acid (IAA). Five out of 16 isolates significantly stimulated shoot and root growth of marigold in a pot experiment. The present study reveals that more than half of the bacterial isolates associated with marigold (*C. officinalis* L.) provided antifungal activity against one or more plant pathogenic fungi. Our findings suggest that medicinal plants with antimicrobial activity could be a source for selecting microbes with antagonistic activity against fungal plant pathogens or with plant growth stimulating potential. These isolates might be considered as promising candidates for the improvement of plant health.

## 1. Introduction

Marigold (*Calendula officinalis* L.) belongs to the *Asteraceae* family, a native of Mediterranean countries, and is broadly distributed in Europe and also Asia [1]. The plant is traditionally widely used for the treatment of inflammatory swellings, wounds, skin diseases, oral disease, or urinary disorders [2],[3]. Various biological activities of plant extracts from *C. officinalis* L. were reported, including antimicrobial [4]–[6], antidiabetic and antihyperlipidemic activity [3]. Marigold is cultivated not only for the pharmaceutical industry but also for the food and cosmetics industries in several countries of the world, including Europe and India [3],[7]. However, several pathogenic fungi and bacteria have detrimental impacts on the commercial production of this plant, e.g., flower blight (*Alternaria zinniae*), Botrytis blight (*Botrytis cinerea*), damping-off (*Pythium* sp., *Rhizoctonia solani*), root rot and wilt disease caused by *Fusarium solani, Rhizoctonia solani*, *Fusarium oxysporium* and wilt and stem rot caused by *Phytophthora cryptogea* [8]–[10]. For example, the leaf spot disease caused by *Cercospora calendulae* may harm and destroy about 50% of marigold plants [11].

Several fungicides are in use to control marigold pathogens [12],[13]. Moreover, chemical fertilisers are also widely used for marigold production, e.g., magnesium [14], nitrogen and phosphorus fertilisers [15],[16]. Basically, it is essential to reduce the use of agrochemicals for the cultivation of medicinal plants since they are typically consumed after harvest without being further processed and moreover, used for pharmaceutical purposes [17]. One of the possible eco-friendly technologies to stimulate plant growth and control their disease is to use plant growth-promoting bacterial inoculants [18]–[20]. Especially endophytes, which are located in internal tissues, leaves, fruits, ovules, nodules, stem or seeds of plants, produce secondary metabolites which hold the potential to support plants to survive and flourish in hostile environments [21]–[24]. Endophytes use several mechanisms to support plant health, including the production of phytohormones [25]–[27], 1-aminocyclopropane-1-carboxylate (ACC) deaminase [28], hydrogen cyanide [29],[30] and induced systemic resistance [31]–[33]. The diversity of endophytic microbes associated with medicinal plant species such as *Aloe vera*, *Mentha arvensis*, *Dracaena cochinchinensis*, *Hedycium acuminatum, Armoracia rusticana*, and others, were reported [34]–[36]. Although several studies reported on the phytochemical constituents and biological activity of marigold (*C. officinalis* L.), to date, there have been no reports of endophytes associated with marigold and their biological properties. The report of Kaki et al. [37] characterizes *Bacillus* species from the rhizosphere of marigold, which showed plant beneficial traits, growth stimulation of chickpea, and biological control of *Sclerotonia sclerotiorum*. Mohammadi et al. [38] isolated endophytes from *Calendula officinalis* L. with antimicrobial activity against some human bacterial pathogens. Here, we report endophytic bacteria associated with marigold (*C. officinalis* L.) from the Chatkal Biosphere Reserve of Uzbekistan. The knowledge of the medicinal plant-associated endophytic bacteria and their physiological activities within plant tissue are essential to improve our understanding of the role of endophytes in plant growth and development. In the current study, we aim: (1) to isolate and identify culturable endophytic bacteria associated with *Calendula officinalis* L. from the Chatkal Biosphere Reserve, Uzbekistan by using 16S rRNA gene analysis, and (2) to evaluate the plant beneficial properties of endophytic bacteria.

## 2. Materials and methods

### 2.1. Plant sample collection

Marigold (*C. officinalis* L.) was collected from Chatkal Biosphere Reserve of Uzbekistan in June 2018. The region is considered unique, with many endemic species. The climate is dry, with annual temperatures ranging from 20–25 °C. Six individual plants at a distance of 20–30 m and more were collected as a whole and stored in zip-lock plastic bags using sterile gloves and transported to the laboratory of the National University of Uzbekistan. The root system of plants was carefully separated from shoots, and soil attached to the roots was washed with sterile water.

### 2.2. Isolation of endophytic bacteria

The root and shoot (10 g fresh weight) were separated and sterilized with 99.9% ethanol for 2 minutes and 10% NaClO for 1 min and rinsed with sterile distilled water two times for 5 min [39]. The sterile leaves and roots were ground with a mortar and pestle, and 1 g of each sample was transferred into plastic tubes with 9 mL sterile phosphate-buffered saline (PBS) and then serially diluted (10^1^–10^5^). 100 µl aliquots from the appropriate dilutions were spread on 30% Tryptic Soy Agar (TSA) (BD, Difco Laboratories, Detroit, USA) supplemented with nystatin 50 µg/mL and plates were incubated at 28 °C for four days. After four days, single colonies with different shape, colour and density were picked and transferred by streaking on nutrient agar plates and incubated for the next 96 h to check the purity of the isolates. Visually homological colonies in sizes, shapes and colours were checked under a microscope for purity and used for DNA isolation. In order to test the sterility of the outer surfaces of the plant parts after sterilization with ethanol, we put two uncut pieces of roots and shoots onto TSA media, and the absence of any colonies after 96 h confirmed that sterilization was successful.

### 2.3. DNA isolation

The individual isolates were cultivated in slopes with TSA at 28 °C for 72 h. Subsequently, the colonies were transferred into tubes with Tryptic Soy Broth (TSB Sigma-Aldrich) using a microbiological loop. The isolates were cultivated in TSB at 28 °C for 72 h. The isolation of DNA from bacteria was performed following the method of Dashti et al. [40]. The tubes with bacterial suspension were incubated at 90 °C for 20 min in a Dry Block Heater (IKA Works, Inc., Wilmington, USA) and centrifuged at 12.000 rpm for 5 min. The presence of DNA in the samples was checked using gel electrophoresis and quantified with NanoDrop™ One (Thermo Fisher Scientific Inc., Waltham, USA).

### 2.4. Polymerase chain reaction (PCR)

The DNA extracted from endophytes was used for 16S rRNA gene analysis. The amplification of 16S rRNA genes was conducted by means of PCR using the primers: 27F 5′-GAGTTTGATCCTGGCTCAG-3′ (Sigma-Aldrich, St. Louis, Missouri, USA) and 1492R 5′-GAAAGGAGGTGATCCAGCC-3′ (Sigma-Aldrich, St. Louis, Missouri, USA) [41]. The reaction mixture (25 µL) contained: 1µL of DNA (15–40 ng), 5 µL of 5x One Taq standard reaction buffer (BioLabs, New England); 0.5 µL of 10 mM dNTP mix (Thermo Scientific), 0.5 µL of 10 mM primer 16SF (Merck), 0.5 µL of 10 mM primer 16SR (25 pmol/mL) (Merck), 1 µL of 0.1% bovine serum albumin (TaKaRa Bio Inc., USA), 0.125 µL of One Taq polymerase (BioLabs, New England), 16.375 µL of MQ water. The PCR was conducted using a PTC-200 thermocycler (Bio-Rad Laboratories, USA). The PCR program was as follows: a primary heating step for 30 s at 94 °C, followed by 30 cycles of denaturation for 15 s at 94 °C, annealing for 30 s at 55 °C, and extension for 1.5 min at 68 °C, then followed by the final step for 20 min at 68 °C. The PCR amplified products were checked by electrophoresis using 0.8% agarose gel containing GelRed.

### 2.5. Restriction fragment length polymorphism (RFLP) analysis

To distinguish similar isolates, we performed a RFLP analysis of 16S rRNA genes according to Jinneman et al. [42]. The digested DNA fragments were examined using gel electrophoresis (1% agarose gel). The gel was visualised using a Digital gel imaging system (Gel-Doc XR TM+, Bio-Rad Laboratories, USA). Identical isolates were eliminated, and the rest was sequenced.

### 2.6. Sequencing and phylogenetic analysis

Before sequencing, the PCR products were cleaned using USB^®^ ExoSAP-IT^®^ PCR Product Cleanup Kit (Affymetrix, USB^®^ Products, USA). Sequencing was conducted using ABI PRISM BigDye 3.1 Terminator Cycle Sequencing Ready Reaction Kit (Applied Biosystems, USA) according to the protocol of a manufacturer. Chromas (v. 2.6.5) software was used for the analysis of the received data. Corrected sequences were merged manually using EMBOSS Explorer (http://emboss.bioinformatics.nl/). The identification of sequences was performed using BLAST (Basic Local Alignment Search Tool) and by comparisons with the GenBank nucleotide data bank from the National Centre for Biotechnology Information (NCBI) (http://www.ncbi.nlm.nih.gov/). The ClustalX 2.1 software was used for multiple alignments of all sequences. The received FASTA format file was used to construct the phylogenetic tree. The evolutionary history was concluded using the Neighbor-Joining method [43]. The sum of a tree branch length is 1.03011234. The percentage of replicate trees in which the associated taxa clustered together in the bootstrap test (500 replicates) are shown next to the branches [44]. The tree is drawn to scale, with branch lengths in the same units as those of the evolutionary distances used to infer the phylogenetic tree. The evolutionary distances were computed using the Maximum Composite Likelihood method [45] and are in the units of the number of base substitutions per site. The analysis involved 33 nucleotide sequences (see supplementary material). All positions containing gaps and missing data were deleted. The final dataset contained in total of 1342 positions. Evolutionary analyses were conducted in MEGA6 [46].

### 2.7. Antifungal activities of plant and endophytic bacteria

The bacterial isolates were cultivated in tubes with Tryptic Soy Broth (TSB Sigma-Aldrich) for 72 h at 28 °C. A solvent extraction procedure described in Elissawy et al. [47] was used to extract cell-free supernatant. Plant extraction was performed using the Soxhlet apparatus described in Rojsanga et al. [48] with some modification. The marigold plants were dried at room temperature, and 10 g plant powder were extracted two times with 200 mL of 80% aqueous ethanol solution at 80 °C, 2 h for each time. The extract was filtered, evaporated to dryness, and the residue was cooled in a desiccator for 30 min and then accurately weighed for analysis.

For the measurement of antifungal activity of plant extracts and cell-free suspensions of endophytic bacterial isolates, the plant pathogenic fungi *Fusarium oxysporum*, *F. solani*, and *Rhizoctonia solani* were used. The fungal pathogens were obtained from the Culture Collection of the National University of Uzbekistan. The fungi were cultivated on peptone dextrose agar (PDA) medium at 27 °C within 5–7 days. Agar disks with fungal growth were cut on small squares (with the side size 7 mm) and placed in the center of plates with fresh PDA medium. The plant extracts and cell-free suspensions were transferred into agar wells. The plates were incubated at 27 °C until the fungi entirely covered the control plates without bacteria. The zone of inhibitions was estimated by measuring the diameter of the zone between wells and fungal growth.

### 2.8. In vitro screening for plant beneficial traits

The production of IAA (indole 3-acetic acid) by endophytic isolates was studied using the method described by Bano and Musarrat [49]. The bacteria were grown in TSB medium for 72 h at 28 °C, then transferred to tubes and centrifuged at 13000 x g for 10 min. One milliliter of supernatant was transferred to a fresh tube to which 100 µl of 10 mM orthophosphoric acid and 2 mL of reagent (1 mL of 0.5 M FeCl_3_ in 50 mL of 35% HClO_4_) were added. The IAA production was evaluated following pink colour developed after 30 min in the dark and calculated by using a calibration curve of pure IAA as a standard. The production of HCN by bacterial isolates was measured using the protocol described by Castric [50], and ACC-deaminase activity as described by Lugtenberg et al. [51]. The production of β-1,3-glucanase was determined following the method of Walsh et al. [52] using the substrate lichenan (Sigma-Aldrich, St. Louis, USA) in top agar plates, and a clear zone indicated degradation of the substrate. Protease secretion was revealed by growing strains on TSA/20 amended with skimmed milk to a final concentration 5%. The halo appearing on the first–second day of cultivation around colonies indicated the presence of extracellular protease [53]. The chitinase activity of bacterial isolates was studied as described by Malleswari and Bagyanarayana [54], and lipase activity using the tween lipase indicator assay [55].

### 2.9. Plant growth promotion

Bacteria were grown in Tryptic Soy Broth (TSB Sigma-Aldrich) for 72 h, and bacterial suspensions were adjusted to an optical density at 620 nm of 0.1 (OD620 = 0.1) corresponding to a cell density of about 10^8^ cells/mL. Marigold (*Calendula officinalis* L.) seeds were coated with bacteria by dipping the seeds in bacterial suspensions. Inoculated seedlings were sown in plastic pots (9 cm diameter; 12 cm deep) containing 350 g of soil. The inoculation treatments were set up in a randomized design with ten replications. The pot experiment had two treatments: seeds non-inoculated with bacteria and seeds inoculated with bacteria. Plants were grown at 19–21 °C during the day and 15–17 °C at night, and after 8 weeks, the leaves with stem and root length and dry weight were measured [56].

### 2.10. Statistical analysis

Data were tested for statistical significance by the analysis of variance included in Microsoft Excel 2010 package. Mean comparisons were conducted using the least significant difference (LSD) test (P = 0.05). The mean values of IAA production, antimicrobial activity, and the standard deviation were extracted for each observation.

### 2.11. Accession numbers

The 16S rRNA gene sequences of the endophytic bacteria of *C. officinalis* L. were deposited into GenBank under the accession numbers: MH165349–MH165364.

## 3. Results

### 3.1. Isolation and identification of endophytic bacteria

A total of 42 bacterial isolates were isolated from plant tissues of marigold. The RFLP analysis was conducted to eliminate similar isolates, and as a result, only 18 isolates were obtained: 7 from roots and 11 from shoots (Table 1). The isolates were identified using the BLAST basic local alignment search tool and compared with similar strains from the NCBI GenBank. The 16S rRNA sequences similarities of endophytic bacteria isolated from plant tissue of marigold with sequences from GenBank are shown in Table 1.

All isolates were similar by 98.55–99.86% to those registered in GenBank. The isolated endophytes represent four classes of bacteria: Alphaproteobacteria, Betaproteobacteria, Gammaproteobacteria and Actinobacteria. The most numerous were representatives of the class Gammaproteobacteria (11): FRT2, FRT6, FRT12, FRT26, FSN1, FSN4, FSN5, FST1, FST4, FST5 and FST13. Only three isolates belonged to alphaproteobacteria: FRN6, FRT3 and FRT9. Betaproteobacteria were represented by isolate FST8 and Actinobacteria by isolate FST16.

**Figure 1.**
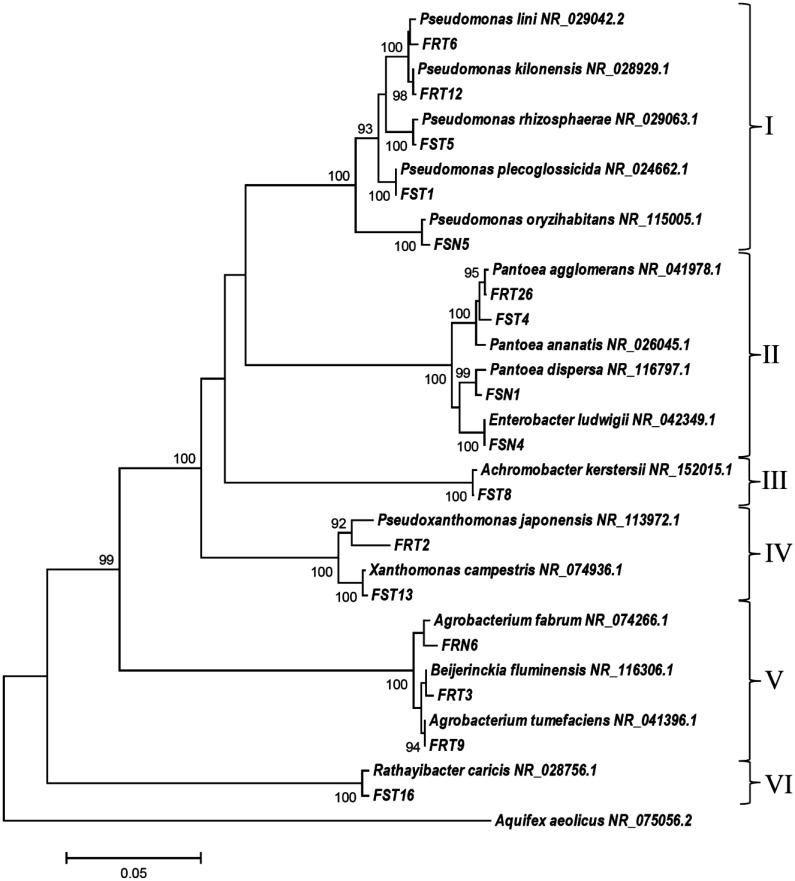
Phylogenetic tree of bacterial endophytes isolated from *Calendula officinalis* L. and their closest relatives from GenBank. I–VI–clusters (orders): I–Pseudomonadales, II–Enterobacteriales, III–Burkholderiales, IV–Xanthomonadales, V–Rhizobiales, VI–Actinomycetales.

A phylogenetic tree using the Neighbor-Joining method was constructed, showing the closest relatives of the isolates (Figure 1).

**Table 1.** Sequence similarities of endophytic bacteria isolated from the roots and shoots of marigold (*C. officinalis* L.) with sequences registered in GenBank.

Isolated strains deposited to GenBank				Closest match (16S rRNA genes) (GenBank)		
Source of isolation	Strain	Length (bp)	Accession number	Reference strains	Accession number	Per cent identity
Roots	FRN6	1387	MH165349	*Agrobacterium fabrum*	NR_074266.1	99.06
	FRT2	1430	MH165350	*Pseudoxanthomonas japonensis*	NR_113972.1	98.56
	FRT3	1381	MH165351	*Beijerinckia fluminensis*	NR_116306.1	98.92
	FRT6	1440	MH165352	*Pseudomonas lini*	NR_029042.2	98.89
	FRT9	1392	MH165353	*Agrobacterium tumefaciens*	NR_041396.1	99.28
	FRT12	1442	MH165354	*Pseudomonas kilonensis*	NR_028929.1	99.38
	FRT26	1450	MH165355	*Pantoea agglomerans*	NR_041978.1	99.04

Shoots	FSN1	1455	MH165356	*Pantoea dispersa*	NR_116797.1	98.90
	FSN4	1444	MH165357	*Enterobacter ludwigii*	NR_042349.1	99.52
	FSN5	1448	MH165358	*Pseudomonas oryzihabitans*	NR_115005.1	99.24
	FST1	1437	MH165359	*Pseudomonas plecoglossicida*	NR_024662.1	99.86
	FST4	1445	MH165360	*Pantoea ananatis*	NR_026045.1	98.55
	FST5	1441	MH165361	*Pseudomonas rhizosphaerae*	NR_029063.1	98.96
	FST8	1436	MH165362	*Achromobacter kerstersii*	NR_152015.1	99.24
	FST13	1443	MH165363	*Xanthomonas campestris*	NR_074936.1	99.38
	FST16	1433	MH165364	*Rathayibacter caricis*	NR_028756.1	98.89
	FRT9	1392	MH165353	*Agrobacterium tumefaciens*	NR_041396.1	99.28
	FRT26	1450	MH165355	*Pantoea agglomerans*	NR_041978.1	99.04

There were six main clusters in the tree. As it is clearly seen by the tree, the most diverse genus (cluster I) from the isolated endophytes is *Pseudomonas* represented by five Gram-negative strains: *Pseudomonas lini* FRT6, *Pseudomonas kilonensis* FRT12, *Pseudomonas oryzihabitans* FSN5, *Pseudomonas plecoglossicida* FST1, *Pseudomonas rhizosphaerae* FST5. Cluster II represented the order of Gram-negative bacteria Enterobacteriales and included the strains *Pantoea agglomerans* FRT26, *Pantoea dispersa* FSN1, *Pantoea ananatis* FST4, and *Enterobacter ludwigii* FSN4. Cluster III presented only one Gram-negative strain, i.e., *Achromobacter kerstersii* FST8. Cluster IV presented two Gram-negative strains of family *Xanthomonadaceae*, i.e., *Pseudoxanthomonas japonensis* FRT2 and *Xanthomonas campestris* FST13. Cluster V presented three Gram-negative strains of the order Rhizobiales, i.e., *Agrobacterium fabrum* FRN6, *Beijerinckia fluminensis* FRT3 and *Agrobacterium tumefaciens* FRT9. Cluster VI presented only the Gram-positive strain *Rathayibacter caricis* FST16 from the order Actinobacteria.

### 3.2. Antifungal activities of plant and endophytic bacteria

Among the bacterial strains, *P. kilonensis* FRT12, and *P. rhizosphaerae* FST5 showed moderate or strong inhibition against three tested plant pathogenic fungi *F. culmorum, F. solani* and *R. solani* (Table 2). Other strains exhibited antifungal activity against two tested fungal pathogens. Plant extract showed antifungal activity against *F. solani* and *R. solani*.

**Table 2.** Antifungal activity of bacterial endophytes from marigold (*C. officinalis* L.) against plant pathogenic fungi.

Treatments with bacterial strains	Growth inhibition zone in diameter (mm)		
	*Fusarium oxysporum*	*Fusarium solani*	*Rhizoctonia solani*
*Achromobacter kerstersii* FST8	-	3 ± 1	7 ± 1
*Agrobacterium fabrum* FRN6	-	-	-
*Agrobacterium tumefaciens* FRT9	-	-	-
*Beijerinckia fluminensis* FRT3	9 ± 1	8 ± 1	-
*Enterobacter ludwigii* FSN4	-	-	-
*Pantoea agglomerans* FRT26	8 ± 1	-	7 ± 1
*Pantoea ananatis* FST4	-	-	-
*Pantoea dispersa* FSN1	12 ± 1	-	10 ± 1
*Pseudomonas kilonensis* FRT12	11 ± 1	12 ± 1	11 ± 1
*Pseudomonas lini* FRT6	-	8 ± 1	-
*Pseudomonas oryzihabitans* FSN5	10 ± 1	-	12 ± 1
*Pseudomonas plecoglossicida* FST1	-	-	-
*Pseudomonas rhizosphaerae* FST5	9 ± 1	10 ± 1	8 ± 1
*Pseudoxanthomonas japonensis* FRT2	-	11 ± 1	-
*Rathayibacter caricis* FST16	-	-	-
*Xanthomonas campestris* FST13	-	-	-
Plant extract	-	3 ± 1	5 ± 1

“-” no formation of inhibition zone

### 3.3. Plant beneficial traits

We characterized several plant growth-promoting traits of endophytes isolated from *C. officinalis* L. The results showed that ten out of sixteen root-associated bacteria produced IAA (Table 3). The highest IAA synthesis was observed in the root-associated bacteria *P. oryzihabitans* FSN5 (8.4 µg/mL) and *P. rhizosphaerae* FST5 (9.2 µg/mL). ACC deaminase production was observed in seven out of sixteen bacterial strains, whereas hydrogen cyanide (HCN) production in five strains only (Table 3). The strains were also checked for fungal cell wall degrading enzymes: chitinase, protease, ß-1,3-glucanase and lipase. The majority of strains produced one or more enzymes. Only two isolates *P. kilonensis* FRT12 and *P. rhizosphaerae* FST5 produced all tested enzymes.

**Table 3.** Plant beneficial traits of endophytic bacteria isolated from marigold (*C. officinalis* L.).

Bacterial strains	Fungal cell wall degrading enzymes				HCN	IAA (µg/mL)	ACC deaminase
	Chitinase	Protease	Glucanase	Lipase			
*Achromobacter kerstersii* FST8	+	+	-	-	-	-	+
*Agrobacterium fabrum* FRN6	-	-	-	-	-	4.2 ± 0.3	-
*Agrobacterium tumefaciens* FRT9	-	-	-	-	-	1.2 ± 0.2	+
*Beijerinckia fluminensis* FRT3	+	+	-	+	+	3.9 ± 0.2	-
*Enterobacter ludwigii* FSN4	-	-	-	-	-	-	+
*Pantoea agglomerans* FRT26	-	+	+	+	+	-	-
*Pantoea ananatis* FST4	-	-	-	-	-	3.4 ± 0.2	-
*Pantoea dispersa* FSN1	-	-	+	+	+	-	-
*Pseudomonas kilonensis* FRT12	+	+	+	+	-	4.5 ± 0.3	-
*Pseudomonas lini* FRT6	+	+	-	-	-	7.8 ± 0.3	+
*Pseudomonas oryzihabitans* FSN5	+	+	-	+	+	8.4 ± 0.3	-
*Pseudomonas plecoglossicida* FST1	-	+	-	-	-	7.1 ± 0.3	+
*Pseudomonas rhizosphaerae* FST5	+	+	+	+	+	9.2 ± 0.3	+
*Pseudoxanthomonas japonensis* FRT2	+	-	-	+	-	7.5 ± 0.3	+
*Rathayibacter caricis* FST16	-	-	-	-	-	-	-
*Xanthomonas campestris* FST13	-	-	-	-	-	-	-

“+” positive. “-” negative

### 3.4. Plant growth promotion

The endophytes were tested for their plant growth promotion abilities (Table 4). The inoculation of marigold seeds with endophytic bacteria increased plant shoot and root dry weight. The strongest growth promotion was observed in the case of *B. fluminensis* FRT3 when the shoot dry weight increased by 28%, and root dry weight by 17% as compared to control plants without inoculation. The shoot and root dry weight were also stimulated by *A. tumefaciens* FRT9, *P. lini* FRT6, *P. oryzihabitans* FSN5, and *P. plecoglossicida* FST1 inoculation up to 25 and 29% compared to control plants, respectively.

**Table 4.** The shoot and root dry weight of marigold (*C. officinalis* L.) when seedlings were inoculated with endophytic bacteria. Plants were grown in pots for 8 weeks.

Treatment	Shoot dry weight (g)	Root dry weight (g)
Control (un-inoculated plants)	1.735 ± 0.11	0.457 ± 0.025
*Achromobacter kerstersii* FST8	1.872 ± 0.11	0.491 ± 0.026
*Agrobacterium fabrum* FRN6	2.015 ± 0.21	0.521 ± 0.059*
*Agrobacterium tumefaciens* FRT9	2.184 ± 0.14*	0.546 ± 0.036*
*Beijerinckia fluminensis* FRT3	2.229 ± 0.18*	0.539 ± 0.054*
*Enterobacter ludwigii* FSN4	1.804 ± 0.11	0.505 ± 0.031
*Pantoea agglomerans* FRT26	1.731 ± 0.12	0.462 ± 0.026
*Pantoea ananatis* FST4	2.043 ± 0.16	0.563 ± 0.045*
*Pantoea dispersa* FSN1	1.657 ± 0.08	0,431 ± 0.021
*Pseudomonas kilonensis* FRT12	2.012 ± 0.26	0.507 ± 0.063
*Pseudomonas lini* FRT6	2.148 ± 0.36*	0.592 ± 0.099*
*Pseudomonas oryzihabitans* FSN5	2.123 ± 0.41*	0.532 ± 0.112*
*Pseudomonas plecoglossicida* FST1	2.108 ± 0.29*	0.553 ± 0.079*
*Pseudomonas rhizosphaerae* FST5	2.001 ± 0.48	0.412 ± 0.127
*Pseudoxanthomonas japonensis* FRT2	1.936 ± 0.35	0.541 ± 0.082*

* Significantly different from the un-inoculated plants at P < 0.05

## 4. Discussion

Endophytic bacteria associated with plants play an essential role in plant health. They produce various beneficial metabolites and thus have been considered as a source of valuable biologically active compounds [23]–[26]. To the best of our knowledge, this is the first report where endophytic bacteria associated with marigold (*C. officinalis* L.) grown in the arid Chatkal Biosphere Reserve, Uzbekistan have been analysed. Profiling of endophytic bacteria isolated from the roots of marigold demonstrated that these included 18 isolates belonging to the genera *Pantoea* (4), *Enterobacter, Pseudomonas* (5), *Achromobacter, Xanthomonas, Rathayibacter, Agrobacterium* (3), *Pseudoxanthomonas*, and *Beijerinckia*. Similar bacterial species were isolated from tissues of other medicinal plants, e.g., *P. kilonensis* from *Lotus corniculatus* [23], *B. frigoritolerans* from *Glycyrrhiza uralensis* [57], *B. fluminensis* from rhizome of *Curcuma longa, E. ludwigii* from *Aloe vera* [58] and *P. ananatis* from *Solanum mauritianum* [59]. Notably, we have observed *A. tumefaciens*, and *P. agglomerans* both in the roots and the shoots of the marigold. However, it has been reported previously that bacteria in plant tissues can migrate from soil to aerial parts of the plant through chemotaxis [60],[61].

Moreover, in our study of marigold, several bacterial endophytes demonstrated antagonistic activity against the plant pathogenic fungi *F. oxysporum*, *F. solani*, and *R. solani*. Higher proportions of endophytes with antifungal properties were previously reported among plant-associated bacteria with *Hypericum perforatum* [62],[63] and *Chelidonium majus* L. [64]. Evidence is available that biologically active compounds of medicinal plants may affect endophytic microbes living inside the plant tissue and their physiological functions [24],[65],[66]. According to Mehanni and Safwat [66], endophytic bacteria may demonstrate similar biological activity and produce metabolites as their hosts. This statement was confirmed by the work of Koberl et al. [24] for endophytic bacteria isolated from the medicinal plants *Matricaria chamomilla, Calendula officinalis*, *Solanum distichum*, and for endophytic bacteria isolated from *Hypericum perforatum*, which showed antifungal activities as their host. Moreover, findings showed that fungal pathogens could be successfully controlled by the use of antagonistic features of endophytic bacteria without causing substantive harm to the host [67]–[70]. For example, endophytes associated with *Monarda citriodora*, which showed antagonistic activity against *Fusarium oxysporum*, *F. redolens* proved biocontrol potential [38]. Several basic mechanisms underlying plant beneficial effects were reported in the literature, including the production of phytohormones, fungi cell wall degrading enzymes, hydrogen cyanide (HCN) production and ACC-deaminase [71]–[73]. Ten out of sixteen bacterial strains produced at least one of four checked fungal cell wall degrading enzymes, i.e., chitinase, protease, ß-1,3-glucanase, and lipase. It is known that bacterial production of fungal cell wall degrading enzymes such as chitinase, which can degrade the integral fungal cell wall component-chitin, protease, degrading fungal proteins, lipase, which can degrade some fungal cell wall-associated lipids, β-1,3-glucanase, which can degrade cell wall carbohydrates, is one of the main mechanisms of plant pathogens suppression [64]. Five out of sixteen bacterial strains demonstrated the production of HCN, a mechanism also involved in the suppression of soil-borne pathogens [74]. Michelsen and Stougaar [75] reported that *Pseudomonas fluorescens* strains which synthesized HCN inhibited the hyphal growth *Rhizoctonia solani* and *Pythium aphanidermatum*. In our study, ten out of sixteen bacterial strains were able to produce IAA and stimulated the growth of root or shoot of marigold seedlings. There are numerous reports on the IAA production by endophytic bacteria associated with other medicinal plants [76]. The endophytic bacteria isolated from *Cassia occidentalis*, stimulated the plant growth of mung bean in pot experiments through its ability to produce IAA [77]. Furthermore, bacterial endophytes synthesized ACC deaminase, which can lower the level of ethylene, a plant stress hormone, through which plant stress tolerance increased [28]. In our study, seven out of sixteen endophytic bacterial isolates were able to produce ACC deaminase. It was reported that bacterial strains *P. putida* TSAU1 and *P. aureantiaca* TSAU22 were capable to synthesize ACC deaminase, stimulated the wheat root system in saline soils [56].

Beneficial effects of PGPR on plant growth of medicinal plants were recorded for *Ocimum basilicum* [78], *Calendula officinalis* [79], *Catharanthus roseus* [80] and *Datura* plants [81]. A significant increase in root and shoot growth of *Galega orientalis* was detected after co-inoculation of *P. trivialis* combined with *R. galegae* [82]. Rasool et al. [83] observed plant growth-promoting potential of root-associated bacteria *B. frigoritolerans*, and *B. aryabhattai* isolated from saffron (*Crocus sativus* L.) that could be a promising source of plant growth stimulants to increase cormlets growth and increase saffron production.

## 5. Conclusions

The present study reveals for the first time the isolation, identification and characterisation of endophytic bacteria from marigold (*C. officinalis* L.) sampled from the Chatkal Biosphere reserve of Uzbekistan. Among the identified endophytic isolates, species belonging to *Agrobacterium*, *Pseudomonas*, and *Pantoea* were obtained. The bacterial strains associated with marigold possessed antifungal activity against plant pathogenic fungi and were able to synthesize chitinase, protease, ß-1,3-glucanase, lipase, HCN, IAA and ACC-deaminase. Twelve out of 16 isolates stimulated shoot and root growth of marigold. Our findings suggest that medicinal plants with antimicrobial activity could be a source for selecting microbes with antagonistic activity against plant fungal pathogens and plant growth stimulators and might thus be considered as promising candidates for the improvement of plant health. However, these findings also show that further research is necessary to resolve the impact of endophytic bacteria with selected PGPR traits on plant growth and controlling fungal diseases in field experiments.
