# Supplementary material for: Diversity and biological activity of culturable endophytic bacteria associated with marigold (Calendula officinalis L.)
Source: AIMS Microbiol. 2021 Sep 13;7(3):336–53. doi: 10.3934/microbiol.2021021 (PMC8500798; doi:10.3934/microbiol.2021021)
Supplement: Supplementary file 1 [file microbiol-07-03-021-s001.pdf]

AIMS Microbiology

AIMS Microbiology, 7(3): 336–353.
DOI: 10.3934/microbiol.2021021
Received: 23 July 2021
Accepted: 09 September 2021
Published: 13 September 2021

http://www.aimspress.com/journal/microbiology

Research article

Diversity and biological activity of culturable endophytic bacteria associated with marigold (*Calendula officinalis* L.)

Vyacheslav Shurigin[1,3], Burak Alaylar[2], Kakhramon Davranov[3], Stephan Wirth[4], Sonoko Dorothea Bellingrath-Kimura[4,5] and Dilfuza Egamberdieva[1,4,]*

[1] Department of Microbiology and Biotechnology, Faculty of Biology, National University of Uzbekistan, 100174, Tashkent, Uzbekistan
[2] Department of Molecular Biology and Genetics, Faculty of Arts and Sciences, Agri Ibrahim Cecen University, 04100, Agri, Turkey
[3] Institute of Microbiology of the Academy of Sciences of the Republic of Uzbekistan, 100128 Tashkent, Uzbekistan
[4] Leibniz Centre for Agricultural Landscape Research (ZALF), 15374 Müncheberg, Germany
[5] Faculty of Life Science, Humboldt University of Berlin, 14195, Berlin, Germany

* **Correspondence:** Email: Dilfuza.Egamberdieva@zalf.de; Tel: +998909143366.

Supplementary material

16S rRNA gene sequences used for constructing a phylogenetic tree of bacterial endophytes isolated from *Calendula officinalis* L. and their closest relatives from GenBank

>FRN6
CATGCAGTCGAACGCCCCGCAGGGGAGTGGCAGACGGGTGAGTAACGCGTGGGAACAT
ACCCTTTCCTGCGGAATAGCTCTGGGAAACTGGAATTAATACCGCATACGCCCTACGGG
GGAAAGATTTATCGGGGAAGGATTGGCCCGCGTTGGATTAGCTAGTTGGTGGGGTAAAG
GCCTACCAAGGCGACGATCCATAGCTGGTCTGAGAGGATGATCAGCCACATTGGGACTG
AGACACGGCCCAAACTCCTACGGGAGGCAGCAGTGGGGAATATTGGACAATGGGCGCA
AGCCTGATCCAGCCATGCCGCGTGAGTGATGAAGGCCTTAGGGTTGTAAAGCTCTTTCA

CCGATGAAGATAATGACGGTAGTCGGAGAAGAAGCCCCGGCTAACTTCGTGCCAGCAG
CCGCGGTAATACGAAGGGGGCTAGCGTTGTTCGGAATTACTGGGCGTAAAGCGCACGTA
GGCGGATATTTAAGTCAGGGGTGAAATCCCGCAGCTCAACTGCGGAACTGCCTTTGATA
CTGGGTATCTTGAGTATGGAAGAGGTAAGTGGAATTCCGAGTGTAGAGGTGAAATTCGT
AGATATTCGGAGGAACACCAGTGGCGAAGGCGGCTTACTGGTCCATTACTGACGCTGAG
GTGCGAAAGCGTGGGGAGCAAACAGGATTAGATACCCTGGTAGTCCACGCCGTAAACG
ATGAATGTTAGCCGTCGGGCAGTATACTGTTCGGGTGGCGCAGCTAACGCATTAAACAT
TCCGCCTGGGGGAGTACGGGTCGCAAGATTAAAACTCAAAGGAATTGACGGGGGCCCG
CACAAGCGGTGGAGCATGTGGTTTAATTCGAAGCAACGCGCAGAACCTTACCAGCTCTT
GACATTCGGGGTATGGGCATTGGAGACGATGTCCTTCAGTTAGGCTGGCCCCAGAACAG
GTGCTGCATGGCTGTCGTCAGCTCGTGTCGTGAGATGTTGGGTTAAGTCCCGCAACGAG
CGCAACCCTCGCCCTTAGTTGCCAGCATTTAGTTGGGCACTCTAAGGGGACTGCCGGTG
ATAAGCCGAGAGGAAGGTGGGGATGACGTCAAGTCCTCATGGCCCTTACGGGCTGGGC
TACACACGTGCTACAATGGTGGTGACAGTGGGCAGCGAGACAGCGATGTCGAGCTAAT
CTCCAAAAGCCATCTCAGTTCGGATTGCACTCTGCAACTCGAGTGCATGAAGTTGGAAT
CGCTAGTAATCGCAGATCAGCATGCTGCGGTGAATACGTTCCCGGGCCTTGTACACACC
GCCCGTCACACCATGGGAGTTGGTTTTACCCGAAGGTAGTGCGCTAACCGCAAGGAGGC
AGCTAACCACGGTAGGGTCAGCGACTGGGGTGAAGTC

>FRT2
GGCAGCACAGGAGAGCTTGCTCTCTGGGTGGCGAGTGGCGGACGGGTGAGGAATACAT
CGGAATCTACCTTGTCGTGGGGGATAACGTAGGGAAACTTACGCTAATACCGCATACGA
CCTTCGGGTGAAAGTGGGGGACCGCAAGGCCTCACGCGATTAGATGAGCCGATGTCGG
ATTAGCTAGTTGGCGGGGTAAAAGCCCACCAAGGCGACGATCCGTAGCTGGTCTGAGA
GGATGATCAGCCACACTGGAACTGAGACACGGTCCAGACTCCTACGGGAGGCAGCAGT
GGGGAATATTGGACAATGGGCGCAAGCCTGATCCAGCCATACCGCGTGGGTGAAGAAG
GCCTTCGGGTTGTAAAGCCCTTTTGTTGGGAAAGAAATCTTGCCGGTTAATACCTGGCG
AGGATGACGGTACCCAAAGAATAAGCACCGGCTAACTTCGTGCCAGCAGCCGCGGTAA
TACGAAGGGTGCAAGCGTTACTCGGAATTACTGGGCGTAAAGCGTGCGTAGGTGGTGGT
TTAAGTCTGCTGTGAAAGCCCTGGGCTCAACCTGGGAATTGCAGTGGATACTGGGTCAC
TAGAGTGTGGTAGAGGGATGCGGAATTTCCGGTGTAGCAGTGAAATGCGTAGAGATCG
GAAGGAACATCCGTGGCGAAGGCGGCATCCTGGGCCAACACTGACACTGAGGCACGAA
AGCGTGGGGAGCAAACAGGATTAGATACCCTGGTAGTCCACGCCCTAAACGATGCGAA
CTGGATGTTGGTGCAACTTGGCACCCAGTATCGAAGCTAACGCGTTAAGTTCGCCGCCT
GGGGAGTACGGTCGCAAGACTGAAACTCAAGGAAATTGACGGGGCCCGCACAAGCGGT
GGAGTATGTGGTTTAATTCGATGCAACGCGAAGAACCTTACCTGGCCTTGACATGTCGC
GAACTTTCCAGAGATGGATTGGTGCCTTCGGGAACGCGAACACAGGTGCTGCATGGCTG
TCGTCAGCTCGTGTCGTGAGATGTTGGGTTAAGTCCCGCAACGAGCGCAACCCTTGTCCT
TAGTTGCCAGCACGTAATGGTGGGAACTCTAAGGAGACCGCCGGTGACAAACCGGAGG
AAGGTGGGGATGACGTCAAGTCATCATGGCCCTTACGGCCAGGGCTACACACGTACTAC
AATGGGAAGGACAGAGGGCTGCAAACCCGCGAGGGCAAGCCAATCCCAGAAACCTTCT
CTCAGTCCGGATTGGAGTCTGCAACTCGACTCCATGAAGTCGGAATCGCTAGTAATCGC
AGATCAGCATTGCTGCGGTGAATACGTTCCCGGGCCTTGTACACACCGCCCGTCACACC

ATGGGAGTTTGTTGCACCAGAAGCAGGTAGCTTAACCTTCGGGAGGGCGCTTGCCACGG
TGTGGCCGATGACTGGGGTGAAGTC

>FRT3
ACATGCAGTCGACGCCCCGCAGGGGAGTGGCAGACGGGTGAGTAACGCGTGGGAACAT
ACCCTTTCCTGCGGAATAGCTCCGGGAAACTGGAATTAATACCGCATACGCCCTACGGG
GGAAAGATTTATCGGGGAAGGATTGGCCCGCGTTGGATTAGCTAGTTGGTGGGGTAAAG
GCCTACCAAGGCGACGATCCATAGCTGGTCTGAGAGGATGATCAGCCACATTGGGACTG
AGACACGGCCCAAACTCCTACGGGAGGCAGCAGTGGGGAATATTGGACAATGGGCGCA
AGCCTGATCCAGCCATGCCGCGTGAGTGATGAAGGCCTTAGGGTTGTAAAGCTCTTTCA
CCGGAGAAGATAATGACGGTATCCGGAGAAGAAGCCCCGGCTAACTTCGTGCCAGCAG
CCGCGGTAATACGAAGGGGGCTAGCGTTGTTCGGAATTACTGGGCGTAAAGCGCACGTA
GGCGGATATTTAAGTCAGGGGTGAAATCCCAGAGCTCAACTCTGGAACTGCCTTTGATA
CTGGGTATCTTGAGTATGGAAGAGGTAAGTGGAATTCCGAGTGTAGAGGTGAAATTCGT
AGATATTCGGAGGAACACCAGTGGCGAAGGCGGCTTACTGGTCCATTACTGACGCTGAG
GTGCGAAAGCGTGGGGGAGCAAACAGGATTAGATACCCTGGGTAGTCCACGCCCGTAA
ACGATGAATGTTAGCCGGTCGGGCAGTATACTGTTCGGGTGGGCGCAGCTTAACGCATT
AAACCATTCCGCCTGGGGGAAGTACGGTCGCAAGATTAAAACTCAAAGGAATTGACGG
GGGCCCGCACAAGCGGTGGAGCATGTGGTTTAATTAGAAGCAACGCGCAGAACCTTAC
CAGCTCTTGACATTCGGGGTATGGGCATTGGAGACGATGTCCTTCAGTTAGGCTGGCCC
CAGAACAGGTGCTGCATGGCTGTCGTCAGCTCGTGTCGTGAGATGTTGGGTTAAGTCCC
GCAACGAGCGCAACCCTCGCCCTTAGTTGCCAGCATTTAGTTGGGCACTCTAAGGGGAC
TGCCGGTGATAAGCCGAGAGGAAGGTGGGGATGACGTCAAGTCCTCATGGCCCTTACG
GGCTGGGCTACACACGTGCTACAATGGTGGTGACAGTGGGCAGCGAGACAGCGATGTC
GAGCTAATCTCCAAAAGCCATCTCAGTTCGGATTGCACTCTGCAACTCGAGTGCATGAA
GTTGGAATCGCTAGTAATCGCAGATCAGCATGCTGCGGTGAATACGTTCCCGGGCCTTG
TACACACCGCCCGTCACACCATGGGAGTTGGTTTTACCCGAAGGTAGTGCGCTAACCGC
AAGGAGGCAGCTAACCATGGTAGGGTCAGCGA

>FRT6
TGCAGTCGAGCGGTAGAGAGGTGCTTGCACCTCTTGAGAGCGGCGGACGGGTGAGTAA
TGCCTAGGAATCTGCCTGGTAGTGGGGGATAACGCTCGGAAACGGACGCTAATACCGCA
TACGTCCTACGGGAGAAAGCAGGGGACCTTCGGGCCTTGCGCTATCAGATGAGCCTAGG
TCGGATTAGCTAGTTGGTGAGGTAATGGCTCACCAAGGCGACGATCCGTAACTGGTCTG
AGAGGATGATCAGTCACACTGGAACTGAGACACGGTCCAGACTCCTACGGGAGGCAGC
AGTGGGGAATATTGGACAATGGGCGAAAGCCTGATCCAGCCATGCCGCGTGTGTGAAG
AAGGTCTTCGGATTGTAAAGCACTTTAAGTTGGGAGGAAGGGCAGTTACCTAATACGTA
ATTGTTTTGACGTTACCGACAGAATAAGCACCGGCTAACTCTGTGCCAGCAGCCGCGGT
AATACAGAGGGTGCAAGCGTTAATCGGAATTACTGGGCGTAAAGCGCGCGTAGGTGGT
TCGTTAAGTTGGATGTGAAAGCCCCGGGCTCAACCTGGGAACTGCATTCAAAACTGTCG
AGCTAGAGTATGGTAGAGGGTGGTGGAATTTCCTGTGTAGCGGTGAAATGCGTAGATAT
AGGAAGGAACACCAGTGGCGAAGGCGACCACCTGGACTGATACTGACACTGAGGTGCG
AAAGCGTGGGGAGCAAACAGGATTAGATACCCTGGTAGTCCACGCCGTAAACGATGTC
AACTAGCCGTTGGGAGCCTTGAGCTCTTAGTGGCGCAGCTAACGCATTAAGTTGACCGC

CTGGGGAGTACGGCCGCAAGGTTAAAACTCAAATGAATTGACGGGGGCCCGCACAAGC
GGGTGGAGCATGTGGGTTTAATTTCGAAGCAACGCGAAGAACCTTACCCAGGGCCTTG
ACATCCAATGAACTTTCCAGAGATGGATTGGTGCCTTCGGGAGCATTGAGACAGGTGCT
GCATGGCTGTCGTCAGCTCGTGTCGTGAGATGTTGGGTTAAGTCCCGTAACGAGCGCAA
CCCTTGTCCTTAGTTACCAGCACGTAATGGTGGGCACTCTAAGGAGACTGCCGGTGACA
AACCGGAGGAAGGTGGGGATGACGTCAAGTCATCATGGCCCTTACGGCCTGGGCTACA
CACGTGCTACAATGGTCGGTACAGAGGGTTGCCAAGCCGCGAGGTGGAGCTAATCCCAC
AAAACCGATCGTAGTCCGGATCGCAGTCTGCAACTCGACTGCGTGAAGTCGGAATCGCT
AGTAATCGCGAATCAGAATGTCGCGGTGAATACGTTCCCGGGCCTTGTACACACCGCCC
GTCACACCATGGGAGTGGGTTGCACCAGAAGTAGCTAGTCTAACCTTCGGGGGGACGGT
TACCACGGTGTGATTCATGACTGGGGTGAAGT

>FRT9
CATGCAGTCGAACGCCCCGCAAGGGTGTAGTGGCAGACGGGTGAGTAACGCGTGGGAA
CATACCCTTTCCTGCGGAATAGCTCCGGGAAACTGGAATTAATACCGCATACGCCCTAC
GGGGGAAAGATTTATCGGGGAAGGATTGGCCCGCGTTGGATTAGCTAGTTGGTGGGGTA
AAGGCCTACCAAGGCGACGATCCATAGCTGGTCTGAGAGGATGATCAGCCACATTGGG
ACTGAGACACGGCCCAAACTCCTACGGGAGGCAGCAGTGGGGAATATTGGACAATGGG
CGCAAGCCTGATCCAGCCATGCCGCGTGAGTGATGAAGGCCTTAGGGTTGTAAAGCTCT
TTCACCGGAGAAGATAATGACGGTATCCGGAGAAGAAGCCCCGGCTAACTTCGTGCCA
GCAGCCGCGGTAATACGAAGGGGGCTAGCGTTGTTCGGAATTACTGGGCGTAAAGCGC
ACGTAGGCGGATATTTAAGTCAGGGGTGAAATCCCAGAGCTCAACTCTGGAACTGCCTT
TGATACTGGGTATCTTGAGTATGGAAGAGGTAAGTGGAATTCCGAGTGTAGAGGTGAAA
TTCGTAGATATTCGGAGGAACACCAGTGGCGAAGGCGGCTTACTGGTCCATTACTGACG
CTGAGGTGCGAAAGCGTGGGGAGCAAACAGGATTAGATACCCTGGTAGTCCACGCCGT
AAACGATGAATGTTAGCCGTCGGGCAGTATACTGTTCGGTGGGCGCAGCTAACGCATTA
AACATTCCCGCCTGGGGAGTACGGTCGCAAGATTAAAACTCAAAGGAAATTTGACGGG
GGCCCGCACAAGCGGTGGAGCATGTGGTTTAATTCGAAGCAACGCGCAGAACCTTA
CCAGCTCTTGACATTCGGGGTTTGGGCAGTGGAGACATTGTCCTTCAGTTAGGCTGGCCC
CAGAACAGGTGCTGCATGGCTGTCGTCAGCTCGTGTCGTGAGATGTTGGGTTAAGTCCC
GCAACGAGCGCAACCCTCGCCCTTAGTTGCCAGCATTTAGTTGGGCACTCTAAGGGGAC
TGCCGGTGATAAGCCGAGAGGAAGGTGGGGATGACGTCAAGTCCTCATGGCCCTTACG
GGCTGGGCTACACACGTGCTACAATGGTGGTACAGTGGGCAGCGAGACAGCGATGTCG
AGCTAATCTCCAAAAGCCATCTCAGTTCGGATTGCACTCTGCAACTCGAGTGCATGAAG
TTGGAATCGCTAGTAATCGCAGATCAGCATGCTGCGGTGAATACGTTCCCGGGCCTTGT
ACACACCGCCCGTCACACCATGGGAGTTGGTTTTACCCGAAGGTAGTGCGCTAACCGCA
AGGAGGCAGCTAACCACGGTAGGGTCAGCGACTGGGGTGAAGTC

>FRT12
ATGCAGTCGAGCGGTAGAGAGGTGCTTGCACCTCTTGAGAGCGGCGGACGGGTGAGTA
ATGCCTAGGAATCTGCCTGGTAGTGGGGGATAACGCTCGGAAACGGACGCTAATACCGC
ATACGTCCTACGGGAGAAAGCAGGGGACCTTCGGGCCTTGCGCTATCAGATGAGCCTAG
GTCGGATTAGCTAGTTGGTGGGGTAATGGCTCACCAAGGCGACGATCCGTAACTGGTCT
GAGAG

GATGATCAGTCACACTGGAACTGAGACACGGTCCAGACTCCTACGGGAGGCAGCAGTG
GGGAATATTGGACAATGGGCGAAAGCCTGATCCAGCCATGCCGCGTGTGTGAAGAAGG
TCTTCGGATTGTAAAGCACTTTAAGTTGGGAGGAAGGGCATTAACCTAATACGTTAGTG
TTTTGACGTTACCGACAGAATAAGCACCGGCTAACTCTGTGCCAGCAGCCGCGGTAATA
CAGAGGGTGCAAGCGTTAATCGGAATTACTGGGCGTAAAGCGCGCGTAGGTGGTTTGTT
AAGTTGGATGTGAAAGCCCCGGGCTCAACCTGGGAACTGCATTCAAAACTGACAAGCTA
GAGTATGGTAGAGGGTGGTGGAATTTCCTGTGTAGCGGTGAAATGCGTAGATATAGGAA
GGAACACCAGTGGCGAAGGCGACCACCTGGACTGATACTGACACTGAGGTGCGAAAGC
GTGGGGAGCAAACAGGATTAGATACCCTGGTAGTCCACGCCGTAAACGATGTCAACTA
GCCGTTGGGAGCCTTGAGCTCTTAGTGGCGCAGCTAACGCATTAAGTTGACCGCCTGGG
GAGTACGGCCGCAAGGTTAAAACTCAAATGAATTGACGGGGGCCCGCACAAGCGGTG
GGAGCATGTGGTTTAATTCGAAGCAACCCGAAGAACCCTTACCAGGCCTTTGACAATC
CAATGAACTTTCCAGAGATGGATTGGTGCCTTCGGGAACATTGAGACAGGTGCTGCATG
GCTGTCGTCAGCTCGTGTCGTGAGATGTTGGGTTAAGTCCCGTAACGAGCGCAACCCTT
GTCCTTAGTTACCAGCACGTCATGGTGGGCACTCTAAGGAGACTGCCGGTGACAAACCG
GAGGAAGGTGGGGATGACGTCAAGTCATCATGGCCCTTACGGCCTGGGCTACACACGTG
CTACAATGGTCGGTACAGAGGGTTGCCAAGCCGCGAGGTGGAGCTAATCCCACAAAAC
CGATCGTAGTCCGGATCGCAGTCTGCAACTCGACTGCGTGAAGTCGGAATCGCTAGTAA
TCGCGAATCAGAATGTCGCGGTGAATACGTTCCCGGGCCTTGTACACACCGCCCGTCAC
ACCATGGGAGTGGGTTGCACCAGAAGTAGCTAGTCTAACCTTCGGGGGGACGGTTACCA
CGGTGTGATTCATGACTGGGGTGAAGTC

>FRT26
CATGCAGTCGGACGGTAGCACAGAGAGCTTGCTCTCGGGTGACGAGTGGCGGACGGGT
GAGTAATGTCTGGGGATCTGCCCGATAGAGGGGGATAACCACTGGAAACGGTGGCTAA
TACCGCATAACGTCGCAAGACCAAAGAGGGGGACCTTCGGGCCTCTCACTATCGGATGA
ACCCAGATGGGATTAGCTAGTAGGCGGGGTAATGGCCCACCTAGGCGACGATCCCTAGC
TGGTCTGAGAGGATGACCAGCCACACTGGAACTGAGACACGGTCCAGACTCCTACGGG
AGGCAGCAGTGGGGAATATTGCACAATGGGCGCAAGCCTGATGCAGCCATGCCGCGTG
TATGAAGAAGGCCTTCGGGTTGTAAAGTACTTTCAGCGGGGAGGAAGGCGATGGGGTT
AATAACCCTGTCGATTGACGTTACCCGCAGAAGAAGCACCGGCTAACTCCGTGCCAGCA
GCCGCGGTAATACGGAGGGTGCAAGCGTTAATCGGAATTACTGGGCGTAAAGCGCACG
CAGGCGGTCTGTTAAGTCAGATGTGAAATCCCCGGGCTTAACCTGGGAACTGCATTTGA
AACTGGCAGGCTTGAGTCTTGTAGAGGGGGGTAGAATTCCAGGTGTAGCGGTGAAATGC
GTAGAGATCTGGAGGAATACCGGTGGCGAAGGCGGCCCCCTGGACAAAGACTGACGCT
CAGGTGCGAAAGCGTGGGGAGCAAACAGGATTAGATACCCTGGTAGTCCACGCCGTAA
ACGATGTCGACTTGGAGGTTGTTCCCTTGAGGAGTGGCTTCCCGGAGCTAACGCGTTAA
GTCGACCGCCCTGGGGAGTACGGCCGCAAGGTTAAAACTCAAATGAATTTGACGGGG
GCCCGCACAAGCGGTGGAGCATGTGGTTTAATTCGATGCAACGCGAAGAACCTTACC
TACTCTTGACATCCAGGGAATTTGGCAGAGATGCTTTAGTGCCTTCGGGAACCGTGAGA
CAGGTGCTGCATGGCTGTCGTCAGCTCGTGTTGTGAAATGTTGGGTTAAGTCCCGCAAC
GAGCGCAACCCTTATCCTTTGTTGCCAGCGATTCGGTCGGGAACTCAAAGGAGACTGCC
GGTGATAAACCGGAGGAAGGTGGGGATGACGTCAAGTCATCATGGCCCTTACGAGTAG
GGCTACACACGTGCTACAATGGCGCATACAAAGAGAAGCGACCTCGCGAGAGCAAGCG

GACCTCACAAAGTGCGTCGTAGTCCGGATCGGAGTCTGCAACTCGACTCCGTGAAGTCG
GAATCGCTAGTAATCGTGGATCAGAATGCCACGGTGAATACGTTCCCGGGCCTTGTACA
CACCGCCCGTCACACCATGGGAGTGGGTTGCAAAAGAAGTAGGTAGCTTAACCTTCGGG
AGGGCGCTTACCACTTTGTGATTCATGACTGGGGTGAAGTCGTAA

>FSN1
TACCATGCAGTCGAGCGGCAGCACAGAAGAGCTTGCTCTTTGGGTGGCGAGTGGCGGAC
GGGTGAGTAATGTCTGGGAAACTGCCCGATGGAGGGGGATAACTACTGGAAACGGTAG
CTAATACCGCATAACGTCGCGAGACCAAAGTGGGGGACCTTCGGGCCTCACACCATCGG
ATGTGCCCAGATGGGATTAGCTAATAGGTGGGGTAATGGCTCACCTAGGCGACGATCCC
TAGCTGGTCTGAGAGGATGACCAGCCACACTGGAACTGAGACACGGTCCAGACTCCTAC
GGGAGGCAGCAGTGGGGAATATTGCACAATGGGCGCAAGCCTGATGCAGCCATGCCGC
GTGTATGAAGAAGGCCTTCGGGTTGTAAAGTACTTTCAGCGGGGAGGAAGGCGGTGAG
GTTAATAACCTCGCCGATTGACGTTACCCGCAGAAGAAGCACCGGCTAACTCCGTGCCA
GCAGCCGCGGTAATACGGAGGGTGCAAGCGTTAATCGGAATTACTGGGCGTAAAGCGC
ACGCAGGCGGTCTGTTAAGTCAGATGTGAAATCCCCGGGCTTAACCTGGGAACTGCATT
TGAAACTGGCAGGCTTGAGTCTCGTAGAGGGGGGTAGAATTCCAGGTGTAGCGGTGAA
ATGCGTAGAGATCTGGAGGAATACCGGTGGCGAAGGCGGCCCCCTGGACGAAGACTGA
CGCTCAGGTGCGAAAGCGTGGGGAGCAAACAGGATTAGATACCCTGGTAGTCCACGCC
GTAAACGATGTCGACTTGGAGGTTGTGCCCTTGAGGCGTGGCTTCCGGGAGCTAACGCG
TTAAGTCGACCGCCTGGGGAGTACGGCCGCAAGGTTAAAACTCAAATGAAATTGACG
GGGGCCCGCACAAGCGGTGGAGCATGTGGTTTAATTCGATGCAACGCGAAGAACC
TTACCTGGCCTTGACATCCAGAGAACTTAGCAGAGATGCTTTGGTGCCTTCGGGAACTCT
GAGACAGGTGCTGCATGGCTGTCGTCAGCTCGTGTTGTGAAATGTTGGGTTAAGTCCCG
CAACGAGCGCAACCCTTATCCTTTGTTGCCAGCGGTTCGGCCGGGAACTCAAAGGAGAC
TGCCGGTGATAAACCGGAGGAAGGTGGGGATGACGTCAAGTCATCATGGCCCTTACGG
CCAGGGCTACACACGTGCTACAATGGCGCATACAAAGAGAAGCGACCTCGCGAGAGCA
AGCGGACCTCATAAAGTGCGTCGTAGTCCGGATTGGAGTCTGCAACTCGACTCCATGAA
GTCGGAATCGCTAGTAATCGTAGATCAGAATGCTACGGTGAATACGTTCCCGGGCCTTG
TACACACCGCCCGTCACACCATGGGAGTGGGTTGCAAAAGAAGTAGGTAGCTTAACCTT
CGGCGAGGGCGCTTACCACTTTGTGATTCATGACTGGGGTGAAGTCGAA

>FSN4
CATGCAGTCGACGGTAGCACAGAGAGCTTGCTCTCGGGTGACGAGTGGCGGACGGGTG
AGTAATGTCTGGGAAACTGCCTGATGGAGGGGGATAACTACTGGAAACGGTAGCTAAT
ACCGCATAACGTCGCAAGACCAAAGAGGGGGACCTTCGGGCCTCTTGCCATCAGATGTG
CCCAGATGGGATTAGCTAGTAGGTGGGGTAACGGCTCACCTAGGCGACGATCCCTAGCT
GGTCTGAGAGGATGACCAGCCACACTGGAACTGAGACACGGTCCAGACTCCTACGGGA
GGCAGCAGTGGGGAATATTGCACAATGGGCGCAAGCCTGATGCAGCCATGCCGCGTGT
ATGAAGAAGGCCTTCGGGTTGTAAAGTACTTTCAGCGGGGAGGAAGGTGTTGCGGTTAA
TAACCGCAGCAATTGACGTTACCCGCAGAAGAAGCACCGGCTAACTCCGTGCCAGCAGC
CGCGGTAATACGGAGGGTGCAAGCGTTAATCGGAATTACTGGGCGTAAAGCGCACGCA
GGCGGTCTGTCAAGTCGGATGTGAAATCCCCGGGCTCAACCTGGGAACTGCATTCGAAA
CTGGCAGGCTAGAGTCTTGTAGAGGGGGGTAGAATTCCAGGTGTAGCGGTGAAATGCGT

AGAGATCTGGAGGAATACCGGTGGCGAAGGCGGCCCCCTGGACAAAGACTGACGCTCA
GGTGCGAAAGCGTGGGGA
GCAAACAGGATTAGATACCCTGGTAGTCCACGCCGTAAACGATGTCGACTTGGAGGTTG
TGCCCTTGAGGCGTGGCTTCCGGAGCTAACGCGTTAAGTCGACCGCCTGGGGAGTACGG
CCGCAAGGTTAAAACTCAAATGAATTTGACGGGGGCCCGCACAAGCGGTGGAGCATG
TGGTTTAATTCGATGCAACGCGAAGAACCTTACCTACTCTTGACATCCAGAGAACTT
AGCAGAGATGCTTTGGTGCCTTCGGGAACTCTGAGACAGGTGCTGCATGGCTGTCGTCA
GCTCGTGTTGTGAAATGTTGGGTTAAGTCCCGCAACGAGCGCAACCCTTATCCTTTGTTG
CCAGCGGTCCGGCCGGGAACTCAAAGGAGACTGCCAGTGATAAACTGGAGGAAGGTGG
GGATGACGTCAAGTCATCATGGCCCTTACGAGTAGGGCTACACACGTGCTACAATGGCG
CATACAAAGAGAAGCGAACTCGCGAGAGCAAGCGGACCTCATAAAGTGCGTCGTAGTC
CGGATTGGAGTCTGCAACTCGACTCCATGAAGTCGGAATCGCTAGTAATCGTAGATCAG
AATGCTACGGTGAATACGTTCCCGGGCCTTGTACACACCGCCCGTCACACCATGGGAGT
GGGTTGCAAAAGAAGTAGGTAGCTTAACCTTCGGGAGGGCGCTTACCACTTTGTGATTC
ATGACTGGGGTGAAGTCG

>FSN5
ACATGCAGTCGAGCGGATGAGAGGAGCTTGCTCTTCGATTCAGCGGCGGACGGGTGAGT
AATGCCTAGGAATCTGCCTAGTAGTGGGGGACAACGTTTCGAAAGGAACGCTAATACCG
CATACGTCCTACGGGAGAAAGTGGGGGATCTTCGGACCTCACGCTATTAGATGAGCCTA
GGTCGGATTAGCTAGTTGGTAGGGTAAAGGCCTACCAAGGCGACGATCCGTAACTGGTC
TGAGAGGATGATCAGTCACACTGGAACTGAGACACGGTCCAGACTCCTACGGGAGGCA
GCAGTGGGGAATATTGGACAATGGGCGAAAGCCTGATCCAGCCATGCCGCGTGTGTGA
AGAAGGCCTTCGGGTCGTAAAGCACTTTAAGTTGGGAGGAAGGGCTCATAGCGAATAC
CTGTGAGTTTTGACGTTACCAACAGAATAAGCACCGGCTAACTTCGTGCCAGCAGCCGC
GGTAATACGAAGGGTGCAAGCGTTAATCGGAATTACTGGGCGTAAAGCGCGCGTAGGT
GGCTTGATAAGTTGGATGTGAAATCCCCGGGCTCAACCTGGGAACTGCATCCAAAACTG
TCTGGCTAGAGTGCGGTAGAGGGTAGTGGAATTTCCAGTGTAGCGGTGAAATGCGTAGA
TATTGGAAGGAACACCAGTGGCGAAGGCGACTACCTGGACTGACACTGACACTGAGGT
GCGAAAGCGTGGGGAGCAAACAGGATTAGATACCCTGGTAGTCCACGCCGTAAACGAT
GTCAACTAGCCGTTGGGATCCTTGAGATCTTAGTGGCGCAGCTAACGCATTTAAGTTGA
CCGCCTGGGGGAGTACGGCCCGCAAGGGTTAAAACTCAATGAAATTGACGGGGGCCCC
GCCACAAGCGGTGGAGCATGTGGTTTAATTCGAAGCAACGCGAAGAACCTTACCTGGCC
TTGACATGCTGAGAACTTTCCAGAGATGGATTGGTGCCTTCGGGAACTCAGACACAGGT
GCTGCATGGCTGTCGTCAGCTCGTGTCGTGAGATGTTGGGTTAAGTCCCGTAACGAGCG
CAACCCTTGTCCTTAGTTACCAGCACGTTATGGTGGGCACTCTAAGGAGACTGCCGGTG
ACAAACCGGAGGAAGGTGGGGATGACGTCAAGTCATCATGGCCCTTACGGCCAGGGCT
ACACACGTGCTACAATGGTCGGTACAAAGGGTTGCCAAGCCGCGAGGTGGAGCTAATC
CCATAAAACCGATCGTAGTCCGGATCGCAGTCTGCAACTCGACTGCGTGAAGTCGGAAT
CGCTAGTAATCGTGAATCAGAACGTCACGGTGAATACGTTCCCGGGCCTTGTACACACC
GCCCGTCACACCATGGGAGTGGGTTGCTCCAGAAGTAGCTAGTCTAACCTTCGGGGGGA
CGGTTACCACGGAGTGATTCATGACTGGGGTGAAGTCGTAA

>FST1
CATGCAGTCGAGCGGATGACGGGAGCTTGCTCCTTGATTCAGCGGCGGACGGGTGAGTA
ATGCCTAGGAATCTGCCTGGTAGTGGGGGACAACGTTTCGAAAGGAACGCTAATACCGC
ATACGTCCTACGGGAGAAAGCAGGGGACCTTCGGGCCTTGCGCTATCAGATGAGCCTAG
GTCGGATTAGCTAGTTGGTGGGGTAATGGCTCACCAAGGCGACGATCCGTAACTGGTCT
GAGAGGATGATCAGTCACACTGGAACTGAGACACGGTCCAGACTCCTACGGGAGGCAG
CAGTGGGGAATATTGGACAATGGGCGAAAGCCTGATCCAGCCATGCCGCGTGTGTGAA
GAAGGTCTTCGGATTGTAAAGCACTTTAAGTTGGGAGGAAGGGCAGTAAGCTAATACCT
TGCTGTTTTGACGTTACCGACAGAATAAGCACCGGCTAACTCTGTGCCAGCAGCCGCGG
TAATACAGAGGGTGCAAGCGTTAATCGGAATTACTGGGCGTAAAGCGCGCGTAGGTGG
TTCGTTAAGTTGGATGTGAAAGCCCCGGGCTCAACCTGGGAACTGCATCCAAAACTGGC
GAGCTAGAGTACGGTAGAGGGTGGTGGAATTTCCTGTGTAGCGGTGAAATGCGTAGATA
TAGGAAGGAACACCAGTGGCGAAGGCGACCACCTGGACTGATACTGACACTGAGGTGC
GAAAGCGTGGGGAGCAAACAGGATTAGATACCCTGGTAGTCCACGCCGTAAACGATGT
CAACTAGCCGTTGGAATCCTTGAGATTTTAGTGGCGCAGCTAACGCATTAAGTTGACCG
CCTGGGGAGTACGGCCGCAAGGTTAAAACTCAAATGAATTGACGGGGGCCCGCACAAG
CGGGTGGAGCATGTGGTTTAATTCGAAGCAACGCGAAGAACCTTACCAGGCCTTGACAT
GCAGAGAACTTTCCAGAGATGGATTGGTGCCTTCGGGAACTCTGACACAGGTGCTGCAT
GGCTGTCGTCAGCTCGTGTCGTGAGATGTTGGGTTAAGTCCCGTAACGAGCGCAACCCT
TGTCCTTAGTTACCAGCACGTTATGGTGGGCACTCTAAGGAGACTGCCGGTGACAAACC
GGAGGAAGGTGGGGATGACGTCAAGTCATCATGGCCCTTACGGCCTGGGCTACACACGT
GCTACAATGGTCGGTACAGAGGGTTGCCAAGCCGCGAGGTGGAGCTAATCTCACAAAA
CCGATCGTAGTCCGGATCGCAGTCTGCAACTCGACTGCGTGAAGTCGGAATCGCTAGTA
ATCGCGAATCAGAATGTCGCGGTGAATACGTTCCCGGGCCTTGTACACACCGCCCGTCA
CACCATGGGAGTGGGTTGCACCAGAAGTAGCTAGTCTAACCTTCGGGAGGACGGTTACC
ACGGTGTGATTCATGACTGGGGTGAAGT

>FST4
TGCAGTCGGACGGTAGCACAGAGAGACTTGCTCTCGGGTGACGAGTGGCGGACGGGTG
AGTAATGTCTGGGGATCTGCCCGATAGAGGGGGATAACCACTGGAAACGGTGGCTAAT
ACCGCATAACGTCGCAAGACCAAAGAGGGGGACCTTCGGGCCTCTCACTATCGGATGA
ACCCAGATGGGATTAGCTAGTAGGCGGGGTAATGGCCCACCTAGGCGACGATCCCTAGC
TGGTCTGAGAGGATGACCAGCCACACTGGAACTGAGACACGGTCCAGACTCCTACGGG
AGGCAGCAGTGGGGAATATTGCACAATGGGCGCAAGCCTGATGCAGCCATGCCGCGTG
TATGAAGAAGGCCTTCGGGTTGTAAAGTACTTTCAGCGGGGAGGAAGGCGATGGGGTT
AATAACCCCGTCGATTGACGTTACCCGCAGAAGAAGCACCGGCTAACTCCGTGCCAGCA
GCCGCGGTAATACGGAGGGTGCAAGCGTTAATCGGAATTACTGGGCGTAAAGCGCACG
CAGGCGGTCTGTTAAGTCAGATGTGAAATCCCCGGGCTTAACCTGGGAACTGCATTTGA
AACTGGCAGGCTTGAGTCTTGTAGAGGGGGGTAGAATTCCAGGTGTAGCGGTGAAATGC
GTAGAGATCTGGAGGAATACCGGTGGCGAAGGCGGCCCCCTGGACAAAGACTGACGCT
CAGGTGCGAAAGCGTGGGGAGCAAACAGGATTAGATACCCTGGTAGTCCACGCCGTA
AACGATGTCGACTTGGAGGTTGTTCCCTTGAGGAGTGGCTTCCGGAGCTAACGCGTTAA
GTCGACCGCCCTGGGGAGTACGGCGCAAGGGTTTAAAACTCAAATGAATTTGACGGGG
GCCCCGCACAAGCGGTGGGAGCATGTGGTTTAATTCGATGCAACGCGAAGAACCTTACC

TACTCTTGACATCCAGCGAACTTAGCAGAGATGCTTTGGTGCCTTCGGGAACGCTGAGA
CAGGTGCTGCATGGCTGTCGTCAGCTCGTGTTGTGAAATGTTGGGTTAAGTCCCGCAAC
GAGCGCAACCCTTATCCTTTGTTGCCAGCGATTCGGTCGGGAACTCAAAGGAGACTGCC
GGTGATAAACCGGAGGAAGGTGGGGATGACGTCAAGTCATCATGGCCCTTACGAGTAG
GGCTACACACGTGCTACAATGGCGCATACAAAGAGAAGCGACCTCGCGAGAGCAAGCG
GACCTCACAAAGTGCGTCGTAGTCCGGATCGGAGTCTGCAACTCGACTCCGTGAAGTCG
GAATCGCTAGTAATCGTGGATCAGAATGCCACGGTGAATACGTTCCCGGGCCTTGTACA
CACCGCCCGTCACACCATGGGAGTGGGTTGCAAAAGAAGTAGGTAGCTTAACCTTCGGG
AGGGCGCTTACCACTTTGTGATTCATGACTGGGGTGAAGTC

>FST5
ATGCAGTCGAGCGGTTGAGGAAGCTTGCTTCCTGATTCAGCGGCGGACGGGTGAGTAAT
GCCTAGGAATCTGCCTGGTAGTGGGGGACAACGTTTCGAAAGGAACGCTAATACCGCAT
ACGTCCTACGGGAGAAAGCAGGGGACCTTCGGGCCTTGCGCTATCAGATGAGCCTAGGT
CGGATTAGCTAGTTGGTGAGGTAATGGCTCACCAAGGCGACGATCCGTAACTGGTCTGA
GAGGATGATCAGTCACACTGGAACTGAGACACGGTCCAGACTCCTACGGGAGGCAGCA
GTGGGGAATATTGGACAATGGGCGAAAGCCTGATCCAGCCATGCCGCGTGTGTGAAGA
AGGTCTTCGGATTGTAAAGCACTTTAAGTTGGGAGGAAGGGCAGTCAGCGAATACCTTG
CTGTCTTGACGTTACCGACAGAATAAGCACCGGCTAACTCTGTGCCAGCAGCCGCGGTA
ATACAGAGGGTGCAAGCGTTAATCGGAATTACTGGGCGTAAAGCGCGCGTAGGTGGTTT
GTTAAGTTGAATGTGAAATCCCCGGGCTCAACCTGGGAACTGCATCCAAAACTGGCAAG
CTAGAGTAGGGCAGAGGGTGGTGGAATTTCCTGTGTAGCGGTGAAATGCGTAGATATAG
GAAGGAACACCAGTGGCGAAGGCGACCACCTGGGCTCATACTGACACTGAGGTGCGAA
AGCGTGGGGAGCAAACAGGATTAGATACCCTGGTAGTCCACGCCGTAAACGATGTCAA
CTAGCCGTTGGGAGTCTTGAACTCTTAGTGGCGCAGCTAACGCATTAAGTTGACCGCCT
GGGGAGTACGGGCCGCAAGGTTAAAACTCAAATGAATTTGACGGGGGCCCGCCAC
AAGCGGTGGAGCATGTGGTTAATTCGAAGCAACGCGAAGAACCTTACCAGGCCTTGAC
ATCCAATGAACTTTCCAGAGATGGATTGGTGCCTTCGGGAACATTGAGACAGGTGCTGC
ATGGCTGTCGTCAGCTCGTGTCGTGAGATGTTGGGTTAAGTCCCGTAACGAGCGCAACC
CTTGTCCTTAGTTACCAGCACGTTAAGGTGGGCACTCTAAGGAGACTGCCGGTGACAAA
CCGGAGGAAGGTGGGGATGACGTCAAGTCATCATGGCCCTTACGGCCTGGGCTACACAC
GTGCTACAATGGTCGGTACAGAGGGTTGCCAAGCCGCGAGGTGGAGCTAATCTCACAA
AACCGATCGTAGTCCGGATCGCAGTCTGCAACTCGACTGCGTGAAGTCGGAATCGCTAG
TAATCGCGAATCAGAATGTCGCGGTGAATACGTTCCCGGGCCTTGTACACACCGCCCGT
CACACCATGGGAGTGGGTTGCACCAGAAGTAGCTAGTCTAACCTTCGGGAGGACGGTTA
CCACGGTGTGATTCATGACTGGGGTGAAGTCG

>FST8
TGCAGTCCACGGCAGCACGGACTTCGGTCTGGTGGCGAGTGGCGAACGGGTGAGTAAT
GTATCGGAACGTGCCTAGTAGCGGGGGATAACTACGCGAAAGCGTAGCTAATACCGCA
TACGCCCTACGGGGGAAAGCAGGGGATCGCAAGACCTTGCACTATTAGAGCGGCCGAT
ATCGGATTAGCTAGTTGGTGGGGTAATGGCTCACCAAGGCGACGATCCGTAGCTGGTTT
GAGAGGACGACCAGCCACACTGGGACTGAGACACGGCCCAGACTCCTACGGGAGGCAG
CAGTGGGGAATTTTGGACAATGGGGGAAACCCTGATCCAGCCATCCCGCGTGTGCGATG

AAGGCCTTCGGGTTGTAAAGCACTTTTGGCAGGAAAGAAACGTCATGGGCTAATACCCC
GTGAAACTGACGGTACCTGCAGAATAAGCACCGGCTAACTACGTGCCAGCAGCCGCGG
TAATACGTAGGGTGCAAGCGTTAATCGGAATTACTGGGCGTAAAGCGTGCGCAGGCGGT
TCGGAAAGAAAGATGTGAAATCCCAGAGCTTAACTTTGGAACTGCATTTTTAACTACCG
AGCTAGAGTGTGTCAGAGGGAGGTGGAATTCCGCGTGTAGCAGTGAAATGCGTAGATA
TGCGGAGGAACACCGATGGCGAAGGCAGCCTCCTGGGATAACACTGACGCTCATGCAC
GAAAGCGTGGGGAGCAAACAGGATTAGATACCCTGGTAGTCCACGCCCTAAACGATGT
CAACTAGCTGTTGGGGCCTTCGGGCCTTGGTAGCGCAGCTAACGCGTGAAGTTGACCG
CCTGGGGAGTACGGTCGCAAGATTAAAACTCAAAGGAATTGACGGGGACCCGCACAAG
CGGTGGATGATGTGGATTAATTTCGATGCAACGCGAAAAACCCTTACCTACCCTTTGAC
ATGTCTGGAAATTCCGAAGAGATTTGGAAGTGCTCGCAAGAGAACCGGAACACAGGTG
CTGCATGGCTGTCGTCAGCTCGTGTCGTGAGATGTTGGGTTAAGTCCCGCAACGAGCGC
AACCCTTGTCATTAGTTGCTACGAAAGGGCACTCTAATGAGACTGCCGGTGACAAACCG
GAGGAAGGTGGGGATGACGTCAAGTCCTCATGGCCCTTATGGGTAGGGCTTCACACGTC
ATACAATGGTCGGGACAGAGGGTCGCCAACCCGCGAGGGGGAGCCAATCCCAGAAACC
CGATCGTAGTCCGGATCGCAGTCTGCAACTCGACTGCGTGAAGTCGGAATCGCTAGTAA
TCGCGGATCAGCATGTCGCGGTGAATACGTTCCCGGGTCTTGTACACACCGCCCGTCAC
ACCATGGGAGTGGGTTTTACCAGAAGTAGTTAGCCTAACCGTAAGGGGGGCGATTACCA
CGGTAGGATTCATGACTGGGGTGAAGTCGTA

>FST13
TGCAGTCGACGGCAGCACAGTAAGAGCTTGCTCTTATGGGTGGCGAGTGGCGGACGGGT
GAGGAATACATCGGAATCTACTCTTTCGTGGGGGATAACGTAGGGAAACTTACGCTAAT
ACCGCATACGACCTACGGGTGAAAGCGGAGGACCTTCGGGCTTCGCGCGATTGAATGA
GCCGATGTCGGATTAGCTAGTTGGCGGGGTAAAGGCCCACCAAGGCGACGATCCGTAG
CTGGTCTGAGAGGATGATCAGCCACACTGGAACTGAGACACGGTCCAGACTCCTACGGG
AGGCAGCAGTGGGGAATATTGGACAATGGGCGCAAGCCTGATCCAGCCATGCCGCGTG
GGTGAAGAAGGCCTTCGGGTTGTAAAGCCCTTTTGTTGGGAAAGAAAAGCAGTCGGTTA
ATACCCGATTGTTCTGACGGTACCCAAAGAATAAGCACCGGCTAACTTCGTGCCAGCAG
CCGCGGTAATACGAAGGGTGCAAGCGTTACTCGGAATTACTGGGCGTAAAGCGTGCGTA
GGTGGTGATTTAAGTCTGTTGTGAAAGCCCTGGGCTCAACCTGGGAATTGCAGTGGATA
CTGGGTCACTAGAGTGTGGTAGAGGGTAGCGGAATTCCCGGTGTAGCAGTGAAATGCGT
AGAGATCGGGAGGAACATCCGTGGCGAAGGCGGCTACCTGGACCAACACTGACACTGA
GGCACGAAAGCGTGGGGAGCAAACAGGATTAGATACCCTGGTAGTCCACGCCCTAAAC
GATGCGAACTGGATGTTGGGTGCAATTTGGCACGCAGTATCGAAGCTAACGCGTTAAGT
TCGCCGCCTGGGAGTACGGTCGCAAGACTGAAACTCAAAGGAATTGACGGGGGCCCGC
ACAAGCGGTGAGTATGTGGGTTTAATTCGATGCTACCGCGAAGAACCTTACCTGGTCTT
GACATCCACGGAACTTTCCAGAGATGGATTGGTGCCTTCGGGAACCGTGAGACAGGTGC
TGCATGGCTGTCGTCAGCTCGTGTCGTGAGATGTTGGGTTAAGTCCCGCAACGAGCGCA
ACCCTTGTCCTTAGTTGCCAGCACGTAATGGTGGGAACTCTAAGGAGACCGCCGGTGAC
AAACCGGAGGAAGGTGGGGATGACGTCAAGTCATCATGGCCCTTACGACCAGGGCTAC
ACACGTACTACAATGGTGGGGACAGAGGGCTGCAAACCCGCGAGGGTAAGCCAATCCC
AGAAACCCTATCTCAGTCCGGATTGGAGTCTGCAACTCGACTCCATGAAGTCGGAATCG
CTAGTAATCGCAGATCAGCATTGCTGCGGT

GAATACGTTCCCGGGCCTTGTACACACCGCCCGTCACACCATGGGAGTTTGTTGCACCA
GAAGCAGGTAGCTTAACCTTCGGGAGGGCGCTTGCCACGGTGTGGCCGATGACTGGGGT
GAAGT

>FST16
CATGCAGTCGACGATGAGCCCAGCTTGCTGGGTGGATTAGTGGCGAACGGGTGAGTAAC
ACGTGAGTAACCTGCCCTTGACTCTGGGATAAGCGCTGGAAACGGCGTCTAATACCGGA
TACGACCTGCCCCGGCATCGGGTGCGGGTGGAAAGTTTTTCGGTCAAGGATGGACTCGC
GGCCTATCAGCTTGTTGGTGAGGTAATGGCTCACCAAGGCGACGACGGGTAGCCGGCCT
GAGAGGGTGACCGGCCACACTGGGACTGAGACACGGCCCAGACTCCTACGGGAGGCAG
CAGTGGGGAATATTGCACAATGGGCGAAAGCCTGATGCAGCAACGCCGCGTGGGGGAT
GACGGCCTTCGGGTTGTAAACCTCTTTTAGTAGGGAAGAAGGACTTCGGTTTGACGGTA
CCTGCAGAAAAAGCACCGGCTAACTACGTGCCAGCAGCCGCGGTAATACGTAGGGTGC
AAGCGTTGTCCGGAATTATTGGGCGTAAAGAGCTCGTAGGCGGTTTGTCGCGTCTGCTG
TGAAAACCCGAGGCTCAACCTCGGGCCTGCAGTGGGTACGGGCAGACTAGAGTGCGGT
AGGGGAGAATGGAATTCCTGGTGTAGCGGTGGAATGCGCAGATATCAGGAGGAACACC
GATGGCGAAGGCAGTTCTCTGGGCCGTTACTGACGCTGAGGAGCGAAAGCGTGGGGAG
CGAACAGGATTAGATACCCTGGTAGTCCACGCCGTAAACGTTGGGAACTAGATGTGGGG
GACCTTTCCACGGTCTCCGTGTCGCAGCTAACGCATTAAGTTCCCCGCCTGGGGAGTAC
GGCCGCAAGGCTAAAACTCAAAGGAAATTGACGGGGGCCCGCACAAGCGGCGGAGCA
TGCGGGATTAAATTCGATGCAACGCGGAAGAACCTTACCAAGGCTTTGACATATACC
GGAAACTTCCAGAAATGGTTGCCCCGCAAGGTCGGTATACAGGTGGTGCATGGTTGTCG
TCAGCTCGTGTCGTGAGATGTTGGGTTAAGTCCCGCAACGAGCGCAACCCTCGTTCTAT
GTTGCCAGCACGTCATGGTGGGAACTCATAGGAGACTGCCGGGGTCAACTCGGAGGAA
GGTGGGGATGACGTCAAATCATCATGCCCCTTATGTCTTGGGCTTCACGCATGCTACAAT
GGCCGGTACAAAGGGCTGCGATACCGTAAGGTGGAGCGAATCCCAAAAAGCCGGTCTC
AGTTCGGATTGAGGTCTGCAACTCGACCTCATGAAGTCGGAGTCGCTAGTAATCGCAGA
TCAGCAACGCTGCGGTGAATACGTTCCCGGGCCTTGTACACACCGCCCGTCAAGTCATG
AAAGTCGGTAACACCCGAAGCCGGTGGCCTAACCCCTTGTGGGAGGGAGCCGTCGAAG
GTGGGATCGGTGATTAGGACTAAGTC

>NR_074266.1 *Agrobacterium fabrum*
TTGAGAGTTTGATCCTGGCTCAGAACGAACGCTGGCGGCAGGCTTAACACATGCAAGTC
GAACGCCCCGCAAGGGGAGTGGCAGACGGGTGAGTAACGCGTGGGAATCTACCCATCT
CTGCGGAATAGCTCTGGGAAACTGGAATTAATACCGCATACGCCCTACGGGGGAAAGA
TTTATCGGGGATGGATGAGCCCGCGTTGGATTAGCTAGTTGGTGGGGTAAAGGCCTACC
AAGGCGACGATCCATAGCTGGTCTGAGAGGATGATCAGCCACATTGGGACTGAGACAC
GGCCCAAACTCCTACGGGAGGCAGCAGTGGGGAATATTGGACAATGGGCGCAAGCCTG
ATCCAGCCATGCCGCGTGAGTGATGAAGGCCTTAGGGTTGTAAAGCTCTTTCACCGATG
AAGATAATGACGGTAGTCGGAGAAGAAGCCCCGGCTAACTTCGTGCCAGCAGCCGCGG
TAATACGAAGGGGGCTAGCGTTGTTCGGAATTACTGGGCGTAAAGCGCACGTAGGCGG
ATATTTAAGTCAGGGGTGAAATCCCGCAGCTCAACTGCGGAACTGCCTTTGATACTGGG
TATCTTGAGTATGGAAGAGGTAAGTGGAATTCCGAGTGTAGAGGTGAAATTCGTAGATA
TTCGGAGGAACACCAGTGGCGAAGGCGGCTTACTGGTCCATTACTGACGCTGAGGTGCG

AAAGCGTGGGGAGCAAACAGGATTAGATACCCTGGTAGTCCACGCCGTAAACGATGAA
TGTTAGCCGTCGGGCAGTATACTGTTCGGTGGCGCAGCTAACGCATTAAACATTCCGCC
TGGGGAGTACGGTCGCAAGATTAAAACTCAAAGGAATTGACGGGGGCCCGCACAAGCG
GTGGAGCATGTGGTTTAATTCGAAGCAACGCGCAGAACCTTACCAGCTCTTGACATTCG
GGGTATGGGCATTGGAGACGATGTCCTTCAGTTAGGCTGGCCCCAGAACAGGTGCTGCA
TGGCTGTCGTCAGCTCGTGTCGTGAGATGTTGGGTTAAGTCCCGCAACGAGCGCAACCC
TCGCCCTTAGTTGCCAGCATTTAGTTGGGCACTCTAAGGGGACTGCCGGTGATAAGCCG
AGAGGAAGGTGGGGATGACGTCAAGTCCTCATGGCCCTTACGGGCTGGGCTACACACGT
GCTACAATGGTGGTGACAGTGGGCAGCGAGACAGCGATGTCGAGCTAATCTCCAAAAG
CCATCTCAGTTCGGATTGCACTCTGCAACTCGAGTGCATGAAGTTGGAATCGCTAGTAA
TCGCAGATCAGCATGCTGCGGTGAATACGTTCCCGGGCCTTGTACACACCGCCCGTCAC
ACCATGGGAGTTGGTTTTACCCGAAGGTAGTGCGCTAACCGCAAGGAGGCAGCTAACCA
CGGTAGGGTCAGCGACTGGGGTGAAGTCGTAACAAGGTAGCCGTAGGGGAACCTGCGG
CTGGATCACCTCCTT

>NR_113972.1 *Pseudoxanthomonas japonensis*
AGTGAACGCTGGCGGTAGGCCTAACACATGCAAGTCGAACGGCAGCACAGGAGAGCTT
GCTCTCTGGGTGGCGAGTGGCGGACGGGTGAGGAATACATCGGAATCTACCTTTTCGTG
GGGGATAACGTAGGGAAACTTACGCTAATACCGCATACGACCTACGGGTGAAAGTGGG
GGACCGCAAGGCCTCACGCGATTAGATGAGCCGATGTCCGATTAGCTAGTTGGCGGGGT
AATGGCCCACCAAGGCGACGATCGGTAGCTGGTCTGAGAGGATGATCAGCCACACTGG
AACTGAGACACGGTCCAGACTCCTACGGGAGGCAGCAGTGGGGAATATTGGACAATGG
GCGCAAGCCTGATCCAGCCATACCGCGTGGGTGAAGAAGGCCTTCGGGTTGTAAAGCCC
TTTTGTTGGGAAAGAAATCCTGTCGGTTAATAACCGGTGGGGATGACGGTACCCAAAGA
ATAAGCACCGGCTAACTTCGTGCCAGCAGCCGCGGTAATACGAAGGGTGCAAGCGTTAC
TCGGAATTACTGGGCGTAAAGCGTGCGTAGGTGGTGGTTTAAGTCTGCTGTGAAAGCCC
TGGGCTCAACCTGGGAATTGCAGTGGATACTGGATCACTAGAGTGTGGTAGAGGGATGC
GGAATTTCTGGTGTAGCAGTGAAATGCGTAGAGATCAGAAGGAACATCCGTGGCGAAG
GCGGCATCCTGGGCCAACACTGACACTGAGGCACGAAAGCGTGGGGAGCAAACAGGAT
TAGATACCCTGGTAGTCCACGCCCTAAACGATGCGAACTGGATGTTGGGTGCAACTTGG
CACCCAGTATCGAAGCTAACGCGTTAAGTTCGCCGCCTGGGGAGTACGGTCGCAAGACT
GAAACTCAAAGGAATTGACGGGGGCCCGCACAAGCGGTGGAGTATGTGGTTTAATTCG
ATGCAACGCGAAGAACCTTACCTGGTCTTGACATCCACGGAACTTTCCAGAGATGGATT
GGTGCCTTCGGGAACCGTGAGACAGGTGCTGCATGGCTGTCGTCAGCTCGTGTCGTGAG
ATGTTGGGTTAAGTCCCGCAACGAGCGCAACCCTTGTCCTTAGTTGCCAGCACGTAATG
GTGGGAACTCTAAGGAGACCGCCGGT
GACAAACCGGAGGAAGGTGGGGATGACGTCAAGTCATCATGGCCCTTACGACCAGGGC
TACACACGTACTACAATGGTAGGGACAGAGGGCTGCAAACCCGCGAGGGTGAGCCAAT
CCCAGAAACCCTATCTCAGTCCGGATTGGAGTCTGCAACTCGACTCCATGAAGTCGGAA
TCGCTAGTAATCGCAGATCAGCATTGCTGCGGTGAATACGTTCCCGGGCCTTGTACACA
CCGCCCGTCACACCATGGGAGTTTGTTGCACCAGAAGCAGGTAGCTTAACCTTCGGGAG
GGCGCTTGCCACGGTGTGGCCGATGACTGGGGTGAAG

>NR_116306.1 *Beijerinckia fluminensis*
AGTTTGATCCTGGCTCAGAACGAACGCTGGCGGCAGGCTTAACACATGCAAGTCGAACG
CCCCGCAAGGGGAGTGGCAGACGGGTGAGTAACGCGTGGGAACATACCCTTTCCTGCG
GAATAGCTCCGGGAAACTGGAATTAATACCGCATACGCCCTACGGGGGAAAGATTTATC
GGGGAAGGATTGGCCCGCGTTGGATTAGCTAGTTGGTGGGGTAAAGGCCTACCAAGGC
GACGATCCATAGCTGGTCTGAGAGGATGATCAGCCACATTGGGACTGAGACACGGCCC
AAACTCCTACGGGAGGCAGCAGTGGGGAATATTGGACAATGGGCGCAAGCCTGATCCA
GCCATGCGCGTGAGTGATGAAGGCCTTAGGGTTGTAAAGCTCTTTCACCGGAGAAGATA
ATGACGGTATCCGGAGAAGAAGCCCCGGCTAACTTCGTGCCAGCAGCCGCGGTAATAC
GAAGGGGGCTAGCGTTGTTCGGAATTACTGGGCGTAAAGCGCACGTAGGCGGATATTTA
AGTCAGGGGTGAAATCCCAGAGCTCAACTCTGGAACTGCCTTTGATACTGGGTATCTTG
AGTATGGAAGAGGTAAGTGGAATTCCGAGTGTAGAGGTGAAATTCGTAGATATTCGGA
GGAACACCAGTGGCGAAGGCGGCTTACTGGTCCATTACTGACGCTGAGGTGCGAAAGC
GTGGGGAGCAAACAGGATTAGATACCCTGGTAGTCCACGCCGTAAACGATGAATGTTA
GCCGTCGGGCAGTATACTGTTCGGTGGCGCAGCTAACGCATTAAACATTCCGCCTGGGG
AGTACGGTCGCAAGATTAAAACTCAAAGGAATTGACGGGGGCCCGCACAAGCGGTGGA
GCATGTGGTTTAATTCGAAGCAACGCGCAGAACCTTACCAGCTCTTGACATTCGGGGTA
TGGGCATTGGAGACGATGTCCTTCAGTTAGGCTGGCCCCAGAACAGGTGCTGCATGGCT
GTCGTCAGCTCGTGTCGTGAGATGTTGGGTTAAGTCCCGCAACGAGCGCAACCCTCGCC
CTTAGTTGCCAGCATTTAGTTGGGCACTCTAAGGGGACTGCCGGTGATAAGCCGAGAGG
AAGGTGGGGATGACGTCAAGTCCTCATGGCCCTTACGGGCTGGGCTACACACGTGCTAC
AATGGTGGTGACAGTGGGCAGCGAGACAGCGATGTCGAGCTAATCTCCAAAAGCCATC
TCAGTTCGGATTGCACTCTGCAACTCGAGTGCATGAAGTTGGAATCGCTAGTAATCGCA
GATCAGCATGCTGCGGTGAATACGTTCCCGGGCCTTGTACACACCGCCCGTCACACCAT
GGGAGTTGGTTTTACCCGAAGGTAGTGCGCTAACCGCAAGGAGGCAGCTAACCACGGT
AGGGTCAGCGACTGGGGTGAAGTCGTAACAAGGTAGCCGTAA

>NR_041396.1 *Agrobacterium tumefaciens*
AGCTGGCGGCAGGCTTAACACATGCAAGTCGAACGCCCCGCAAGGGGAGTGGCAGACG
GGTGAGTAACGCGTGGGAACATACCCTTTCCTGCGGAATAGCTCCGGGAAACTGGAATT
AATACCGCATACGCCCTACGGGGGAAAGATTTATCGGGGAAGGATTGGCCCGCGTTGG
ATTAGCTAGTTGGTGGGGTAAAGGCCTACCAAGGCGACGATCCATAGCTGGTCTGAGAG
GATGATCAGCCACATTGGGACTGAGACACGGCCCAAACTCCTACGGGAGGCAGCAGTG
GGGAATATTGGACAATGGGCGCAAGCCTGATCCAGCCATGCCGCGTGAGTGATGAAGG
CCTTAGGGTTGTAAAGCTCTTTCACCGGAGAAGATAATGACGGTATCCGGAGAAGAAGC
CCCGGCTAACTTCGTGCCAGCAGCCGCGGTAATACGAAGGGGGCTAGCGTTGTTCGGAA
TTACTGGGCGTAAAGCGCACGTAGGCGGATATTTAAGTCAGGGGTGAAATCCCAGAGCT
CAACTCTGGAACTGCCTTTGATACTGGGTATCTTGAGTATGGAAGAGGTAAGTGGAATT
CCGAGTGTAGAGGTGAAATTCGTAGATATTCGGAGGAACACCAGTGGCGAAGGCGGCT
TACTGGTCCATTACTGACGCTGAGGTGCGAAAGCGTGGGGAGCAAACAGGATTAGATA
CCCTGGTAGTCCACGCCGTAAACGATGAATGTTAGCCGTCGGGCAGTATACTGTTCGGT
GGCGCAGCTAACGCATTAAACATTCCGCCTGGGGAGTACGGTCGCAAGATTAAAACTCA
AAGGAATTGACGGGGGCCCGCACAAGCGGTGGAGCATGTGGTTTAATTCGAAGCAACG
CGCAGAACCTTACCAGCTCTTGACATTCGGGGTTTGGGCAGTGGAGACATTGTCCTTCA

GTTAGGCTGGCCCCAGAACAGGTGCTGCATGGCTGTCGTCAGCTCGTGTCGTGAGATGT
TGGGTTAAGTCCCGCAACGAGCGCAACCCTCGCCCTTAGTTGCCAGCATTTAGTTGGGC
ACTCTAAGGGGACTGCCGGTGATAAGCCGAGAGGAAGGTGGGGATGACGTCAAGTCCT
CATGGCCCTTACGGGCTGGGCTACACACGTGCTACAATGGTGGTGACAGTGGGCAGCGA
GACAGCGATGTCGAGCTAATCTCCAAAAGCCATCTCAGTTCGGATTGCACTCTGCAACT
CGAGTGCATGAAGTTGGAATCGCTAGTAATCGCAGATCAGCATGCTGCGGTGAATACGT
TCCCGGGCCTTGTACACACCGCCCGTCACACCATGGGAGTTGGTTTACCCGAAGGTAG
TGCGCTAACCGCAAGGAGGCAGCTAACCACGGTAGGGTCAGCGACTGGGGTGAAGTCG
TAACAAGGTAGCCGTAGGGGAACCTGCGGCTGGATCACCTCCTTTCTAAG

>NR_029042.2 *Pseudomonas lini*
AGAGTTTGATCCTGGCTCAGATTGAACGCTGGCGGCAGGCCTAACACATGCAAGTCGAG
CGGTAGAGAGGNGCTTGCACCTCTTGAGAGCGGCGGACGGGTGAGTAATGCCTAGGAA
TCTGCCTGGTAGTGGGGGATAACGCTCGGAAACGGACGCTAATACCGCATACGTCCTAC
GGGAGAAAGCAGGGGACCTTCGGGCCTTGCGCTATCAGATGAGCCTAGGTCGGATTAG
CTAGTTGGTGAGGTAATGGCTCACCAAGGCGACGATCCGTAACTGGTCTGAGAGGATGA
TCAGTCACACTGGAACTGAGACACGGTCCAGACTCCTACGGGAGGCAGCAGTGGGGAA
TATTGGACAATGGGCGAAAGCCTGATCCAGCCATGCCGCGTGTGTGAAGAAGGTCTTCG
GATTGTAAAGCACTTTAAGTTGGGAGGAAGGGCAGTTACCTAATACGTATCTGTTTTGA
CGTTACCGACAGAATAAGCACCGGCTAACTCTGTGCCAGCAGCCGCGGTAATACAGAG
GGTGCAAGCGTTAATCGGAATTACTGGGCGTAAAGCGCGCGTAGGTGGTTCGTTAAGTT
GGATGTGAAATCCCCGGGCTCAACCTGGGAACTGCATTCAAAACTGACGAGCTAGAGTA
TGGTAGAGGGTGGTGGAATTTCCTGTGTAGCGGTGAAATGCGTAGATATAGGAAGGAA
CACCAGTGGCGAAGGCGACCACCTGGACTGATACTGACACTGAGGTGCGAAAGCGTGG
GGAGCAAACAGGATTAGATACCCTGGTAGTCCACGCCGTAAACGATGTCAACTAGCCGT
TGGGAGCCTTGAGCTCTTAGTGGCGCAGCTAACGCATTAAGTTGACCGCCTGGGGAGTA
CGGCCGCAAGGTTAAAACTCAAATGAATTGACGGGGGCCCGCACAAGCGGTGGAGCAT
GTGGTTTAATTCGAAGCAACGCGAAGAACCTTACCAGGCCTTGACATCCAATGAACTTT
CCAGAGATGGATTGGTGCCTTCGGGAACATTGAGACAGGTGCTGCATGGCTGTCGTCAG
CTCGTGTCGTGAGATGTTGGGTTAAGTCCCGTAACGAGCGCAACCCTTGTCCTTAGTTAC
CAGCACGTCATGGTGGGCACTCTAAGGAGACTGCCGGTGACAAACCGGAGGAAGGTGG
GGATGACGTCAAGTCATCATGGCCCTTACGGCCTGGGCTACACACGTGCTACAATGGTC
GGTACAGAGGGTTGCCAAGCCGCGAGGTGGAGCTAATCCCAGAAAACCGATCGTAGTC
CGGATCGCAGTCTGCAACTCGACTGCGTGAAGTCGGAATCGCTAGTAATCGCGAATCAG
AATGTCGCGGTGAATACGTTCCCGGGCCTTGTACACACCGCCCGTCACACCATGGGAGT
GGGTTGCACCAGAAGTAGCTAGTCTAACCTTCGGGAGGACGGTTACCACGGTGTGATTC
ATGACTGGGGTGAAGTCGTAACAAGGTAGCCGTAGGGGAACCTGCGGCTG

>NR_028929.1 *Pseudomonas kilonensis*
GTTTGATCCTGGCTCAGATTGAACGCTGGCGGCAGGCCTAACACATGCAAGTCGAGCGG
TAGAGAGGTGCTTGCACCTCTTGAGAGCGGCGGACGGGTGAGTAATGCCTAGGAATCTG
CCTGGTAGTGGGGGATAACGCTCGGAAACGGACGCTAATACCGCATACGTCCTACGGG
AGAAAGCAGGGGACCTTCGGGCCTTGCGCTATCAGATGAGCCTAGGTCGGATTAGCTAG
TTGGTGGGGTAATGGCTCACCAAGGCGACGATCCGTAACTGGTCTGAGAGGATGATCAG

TCACACTGGAACTGAGACACGGTCCAGACTCCTACGGGAGGCAGCAGTGGGGAATATT
GGACAATGGGCGAAAGCCTGATCCAGCCATGCCGCGTGTGTGAAGAAGGTCTTCGGATT
GTAAAGCACTTTAAGTTGGGAGGAAGGGCATTAACCTAATACGTTAGTGTTTTGACGTT
ACCGACAGAATAAGCACCGGCTAACTCTGTGCCAGCAGCCGCGGTAATACAGAGGGTG
CAAGCGTTAATCGGAATTACTGGGCGTAAAGCGCGCGTAGGTGGTTTGTTAAGTTGGAT
GTGAAAGCCCCGGGCTCAACCTGGGAACTGCATTCAAAACTGACAAGCTAGAGTATGGT
AGAGGGTGGTGGAATTTCCTGTGTAGCGGTGAAATGCGTAGATATAGGAAGGAACACC
AGTGGCGAAGGCGACCACCTGGACTGATACTGACACTGAGGTGCGAAAGCGTGGGGAG
CAAACAGGATTAGATACCCTGGTAGTCCACGCCGTAAACGATGTCAACTAGCCGTTGGG
AGCCTTGAGCTCTTAGTGGCGCAGCTAACGCATTAAGTTGACCGCCTGGGGAGTACGGC
CGCAAGGTTAAAACTCAAATGAATTGACGGGGGCCCGCACAAGCGGTGGAGCATGTGG
TTTAATTCGAAGCAACGCGAAGAACCTTACCAGGCCTTGACATCCAATGAACTTTCCAG
AGATGGATTGGTGCCTTCGGGAACATTGAGACAGGTGCTGCATGGCTGTCGTCAGCTCG
TGTCGTGAGATGTTGGGTTAAGTCCCGTAACGAGCGCAACCCTTGTCCTTAGTTACCAGC
ACGTAATGGTGGGCACTCTAAGGAGACTGCCGGTGACAAACCGGAGGAAGGTGGGGAT
GACGTCAAGTCATCATGGCCCTTACGGCCTGGGCTACACACGTGCTACAATGGTCGGTA
CAGAGGGTTGCCAAGCCGCGAGGTGGAGCTAATCCCACAAAACCGATCGTAGTCCGGA
TCGCAGTCTGCAACTCGACTGCGTGAAGTCGGAATCGCTAGTAATCGCGAATCAGAATG
TCGCGGTGAATACGTTCCCGGGCCTTGTACACACCGCCCGTCACACCATGGGAGTGGGT
TGCACCAGAAGTAGCTAGTCTAACCTTCGGGGGGACGGTTACCACGGTGTGATTCATGA
CTGGGGTGAAGTCGTAACAAGGTAGCCGTAGGCCACGGTGTGATTCATGACTGGGGTGA
A

>NR_041978.1 *Pantoea agglomerans*
GGCCTAACACATGCAAGTCTGACGGTAGCACAGAGGAGCTTGCTCCTTGGGTGACGAGT
GGCGGACGGGTGAGTAATGTCTGGGGATCTGCCCGATAGAGGGGGATAACCACTGGAA
ACGGTGGCTAATACCGCATAACGTCGCAAGACCAAAGAGGGGGACCTTCGGGCCTCTC
ACTATCGGATGAACCCAGATGGGATTAGCTAGTAGGCGGGGTAATGGCCCACCTAGGC
GACGATCCCTAGCTGGTCTGAGAGGATGACCAGCCACACTGGAACTGAGACACGGTCC
AGACTCCTACGGGAGGCAGCAGTGGGGAATATTGCACAATGGGCGCAAGCCTGATGCA
GCCATGCCGCGTGTATGAAGAAGGCCTTCGGGTTGTAAAGTACTTTCAGCGGGGAGGAA
GGCGATGGGGTTAATAACCTTGTCGATTGACGTTACCCGCAGAAGAAGCACCGGCTAAC
TCCGTGCCAGCAGCCGCGGTAATACGGAGGGTGCAAGCGTTAATCGGAATTACTGGGCG
TAAAGCGCACGCAGGCGGTCTGTTAAGTCAGATGTGAAATCCCCGGGCTTAACCTGGGA
ACTGCATTTGAAACTGGCAGGCTTGAGTCTTGTAGAGGGGGGTAGAATTCCAGGTGTAG
CGGTGAAATGCGTAGAGATCTGGAGGAATACCGGTGGCGAAGGCGGCCCCCTGGACAA
AGACTGACGCTCAGGTGCGAAAGCGTGGGGAGCAAACAGGATTAGATACCCTGGTAGT
CCACGCCGTAAACGATGTCGACTTGGAGGTTGTTCCCTTGAGGAGTGGCTTCCGGAGCT
AACGCGTTAAGTCGACCGCCTGGGGAGTACGGCCGCAAGGTTAAAACTCAAATGAATT
GACGGGGGCCCGCACAAGCGGTGGAGCATGTGGTTTAATTCGATGCAACGCGAAGAAC
CTTACCTACTCTTGACATCCACGGAATTTGGCAGAGATGCCTTAGTGCCTTCGGGAACCG
TGAGACAGGTGCTGCATGGCTGTCGTCAGCTCGTGTTGTGAAATGTTGGGTTAAGTCCC
GCAACGAGCGCAACCCTTATCCTTTGTTGCCAGCGATTCGGTCGGGAACTCAAAGGAGA
CTGCCGGTGATAAACCGGAGGAAGGTGGGGATGACGTCAAGTCATCATGGCCCTTACG

AGTAGGGCTACACACGTGCTACAATGGCGCATACAAAGAGAAGCGACCTCGCGAGAGC
AAGCGGACCTCACAAAGTGCGTCGTAGTCCGGATCGGAGTCTGCAACTCGACTCCGTGA
AGTCGGAATCGCTAGTAATCGTGGATCAGAATGCCACGGTGAATACGTTCCCGGGCCTT
GTACACACCGCCCGTCACACCATGGGAGTGGGTTGCAAAAGAAGTAGGTAGCTTAACCT
TCGGGAGGGCGCTTACCACTTTGTGATTCATGACTGGGGTGAAGTCGTAACAAGGTAAC
CGTAGGGG

>NR_029063.1 *Pseudomonas rhizosphaerae*
TAGAGTTTGATCCTGGCTCAGATTGAACGCTGGCGGCAGGCCTAACACATGCAAGTCGA
GCGGTTGAAGGAAGCTTGCTTCCTGATTCAGCGGCGGACGGGTGAGTAATGCCTAGGAA
TCTGCCTGGTAGTGGGGGACAACGTTTCGAAAGGAACGCTAATACCGCATACGTCCTAC
GGGAGAAAGCAGGGGACCTTCGGGCCTTGCGCTATCAGATGAGCCTAGGTCGGATTAG
CTAGTTGGTGAGGTAATGGCTCACCAAGGCGACGATCCGTAACTGGTCTGAGAGGATGA
TCAGTCACACTGGAACTGAGACACGGTCCAGACTCCTACGGGAGGCAGCAGNGGGGAA
TATTGGACAATGGGCGAAAGCCTGATCCAGCCATGCCGCGTGTGTGAAGAAGGTCTTCG
GATTGTAAAGCACTTTAAGTTGGGAGGAAGGGCAGTAAGCGAATACCTTGCTGTTTTGA
CGTTACCGACAGAATAAGCACCGGCTAACTCTGTGCCAGCAGCCGCGGTAATACAGAG
GGTGCAAGCGTTAATCGGAATTACTGGGCGTAAAGCGCGCGTAGGTGGTTTGTTAAGTT
GAATGTGAAATCCCCGGGCTCAACCTGGGAACTGCATCCAAAACTGGCAAGCTAGAGT
AGGGCAGAGGGTGGTGGAATTTCCTGTGTAGCGGTGAAATGCGTAGATATAGGAAGGA
ACACCAGTGGCGAAGGCGACCACCTGGGCTCATACTGACACTGAGGTGCGAAAGCGTG
GGGAGCAAACAGGATTAGATACCCTGGTAGTCCACGCCGTAAACGATGTCAACTAGCC
GTTGGGAGTCTTGAACTCTTAGTGGCGCAGCTAACGCATTAAGTTGACCGCCTGGGGAG
TACGGCCGCAAGGTTAAAACTCAAATGAATTGACGGGGGCCCGCACAAGCGGTGGAGC
ATGTGGTTTAATTCGAAGCAACGCGAAGAACCTTACCAGGCCTTGACATCCAATGAACT
TTCCAGAGATGGATTGGTGCCTTCGGGAACATTGAGACAGGTGCTGCATGGCTGTCGTC
AGCTCGTGTCGTGAGATGTTGGGTTAAGTCCCGTAACGAGCGCAACCCTTGTCCTTAGTT
ACCAGCACGTTATGGTGGGCACTCTAAGGAGACTGCCGGTGACAAACCGGAGGAAGGT
GGGGATGACGTCAAGTCATCATGGCCCTTACGGCCTGGGCTACACACGTGCTACAATGG
TCGGTACAGAGGGTCGCCAAACCGCGAGGTGGAGCTAATCTCACAAAACCGATCGTAG
TCCGGATCGCAGTCTGCAACTCGACTGCGTGAAGTCGGAATCGCTAGTAATCGCGAATC
AGAATGTCGCGGTGAATACGTTCCCGGGCCTTGTACACACCGCCCGTCACACCATGGGA
GTGGGTTGCACCAGAAGTAGCTAGTCTAACCTTCGGGAGGACGGTTACCACGGTGTGAT
TCATGACTGGGGTGAAGTCGTAACAAGGTAGCCGTAGGGGAACCTGTGGCTGGATCACC
TCCTTA

>NR_028756.1 *Rathayibacter caricis*
GTCGACAGAGTTTGATCCTGGCTCAGGACGAACGCTGGCGGCGTGCTTAACACATGCAA
GTCGAACGATGAAGCCCAGCTTGCTGGGTGGATTAGTGGCGAACGGGTGAGTAACACG
TGAGTAACCTGCCCTTGACTCTGGGATAAGCGCTGGAAACGGCGTTTAATACCGGATAC
GACCTGCCCCGGCATCGGGTGCGGGTGGAAAGTTTTTCGGTCAAGGATGGACTCGCGGC
CTATCAGCTTGTTGGTGAGGTAATGGCTCACCAAGGCGACGACGGGTAGCCGGCGTGAG
AGGGTGACCGGCCACACTGGGACTGAGACACGGCCCAGACTCCTACGGGAGGCAGCAG
TGGGGAATATTGCACAATGGGCGAAAGCCTGATGCAGCAACGCCGCGTGGGGGATGAA

GGCCTTCGGGTTGTAAACCTCTTTTAGTAGGGAAGAAGGACTTCGGTTTGACGGTACCT
GCAGAAAAAGCACCGGCTAACTACGTGCCAGCAGCCGCGGTAATACGTAGGGTGCAAG
CGTTGTCCGGAATTATTGGGCGTAAAGAGCTCGTAGGCGGTTTGTCGCGTCTGCTGTGA
AAACCCGAGGCTCAACCTCGGGCCTGCAGTGGGTACGGGCAGACTAGAGTGCGGTAGG
GGAGAATGGAATTCCTGGTGTAGCGGTGGAATGCGCAGATATCAGGAGGAACACCGAT
GGCGAAGGCAGTTCTCTGGGCCGTTACTGACGCTGAGGAGCGAAAGCGTGGGGAGCGA
ACAGGATTAGATACCCTGGTAGTCCACGCCGTAAACGTTGGGAACTAGATGTGGGGACC
TTTCCACGGTCTCCGTGTCGCAGCTAACGCATTAAGTTCCCCGCCTGGGGAGTACGGCC
GCAAGGCTAAAACTCAAAGGAATTGACGGGGGCCCGCACAAGCGGCGGAGCATGCGGA
TTAATTCGATGCAACGCGAAGAACCTTACCAAGGCTTGACATATACCGGAAACTTCCAG
AAATGGTTGCCCCGCAAGGTCGGTATACAGGTGGTGCATGGTTGTCGTCAGCTCGTGTC
GTGAGATGTTGGGTTAAGTCCCGCAACGAGCGCAACCCTCGTTCTATGTTGCCAGCACG
TCATGGTGGGAACTCATAGGAGACTGCCGGGGTCAACTCGGAGGAAGGTGGGGATGAC
GTCAAATCATCATGCCCCTTATGTCTTGGGCTTCACGCATGCTACAATGGCCGGTACAAA
GGGCTGCGATACCGTAAGGTGGAGCGAATCCCAAAAAGCCGGTCTCAGTTCGGATTGA
GGTCTGCAACTCGACCTCATGAAGTCGGAGTCGCTAGTAATCGCAGATCAGCAACGCTG
CGGTGAATACGTTCCCGGGCCTTGTACACACCGCCCGTCAAGTCATGAAAGTCGGTAAC
ACCCGAAGCCGGTGGCCTAACCCCTTGTGGGANGGAGCCGTCGAAGGTGGGATCGGTG
ATTAGGACTAAGTCGTAACAAGGTAACCGTC

>NR_116797.1 *Pantoea dispersa*
AGAGTTTGATCCTGGCTCAGATTGAACGCTGGCGGCAGGCCTAACACATGCAAGTCGAA
CGGCAGCACAGAAGAGCTTGCTCTTTGGGTGGCGAGTGGCGGACGGGTGAGTAATGTCT
GGGAAACTGCCCGATGGAGGGGGATAACTACTGGAAACGGTAGCTAATACCGCATAAT
GTCGCAAGACCAAAGTGGGGGACCTTCGGGCCTCATACCATCGGATGTGCCCAGATGGG
ATTAGCTAGTAGGTGGGGTAATGGCTCACCTAGGCGACGATCCCTAGCTGGTCTGAGAG
GATGACCAGCCACACTGGAACTGAGACACGGTCCAGACTCCTACGGGAGGCAGCAGTG
GGGAATATTGCACAATGGGCGCAAGCCTGATGCAGCCATGCCGCGTGTATGAAGAAGG
CCTTCGGGTTGTAAAGTACTTTCAGCGGGGAGGAAGGCGGTGAGGTTAATAACCTCGCC
GATTGACGTTACCCGCAGAAGAAGCACCGGCTAACTCTGTGCCAGCAGCCGCGGTAATA
CGGAGGGTGCAAGCGTTAATCGGAATTACTGGGCGTAAAGCGCACGCAGGCGGTCTGTT
AAGTCAGATGTGAAATCCCCGGGCTTAACCTGGGAACTGCATTTGAAACTGGCAGGCTT
GAGTCTCGTAGAGGGGGGTAGAATTCCAGGTGTAGCGGTGAAATGCGTAGAGATCTGG
AGGAATACCGGTGGCGAAGGCGGCCCCCTGGACGAAGACTGACGCTCAGGTGCGAAAG
CGTGGGGAGCAAACAGGATTAGATACCCTGGTAGTCCACGCCGTAAACGATGTCGACTT
GGAGGTTATGCCCTTGAGGCGTGGCTTCCGGAGCTAACGCGTTAAGTCGACCGCCTGGG
GAGTACGGCCGCAAGGTTAAAACTCAAATGAATTGACGGGGGCCCGCACAAGCGGTGG
AGCATGTGGTTTAATTCGATGCGACGCGAAGAACCTTACCTGGCCTTGACATCCAGAGA
ACTTAGCAGAGATGCTTTGGTGCCTTCGGGAACTCTGAGACAGGTGCTGCATGGCTGTC
GTCAGCTCGTGTTGTGAAATGTTGGGTTAAGTCCCGCAACGAGCGCAACCCTTATCCTTT
GTTGCCAGCGGTTCGGCCGGGAACTCAAAGGAGACTGCCGGTGATAAACCGGAGGAAG
GTGGGGATGACGTCAAGTCATCATGGCCCTTACGGCCAGGGCTACACACGTGCTACAAT
GGCGCATACAAAGAGAAGCGACCTCGCGAGAGCAAGCGGACCTCATAAAGTGCGTCGT
AGTCCGGATTGGAGTCTGCAACTCGACTCCATGAAGTCGGAATCGCTAGTAATCGTAGA

TCAGAATGCTACGGTGAATACGTTCCCGGGCCTTGTACACACCGCCCGTCACACCATGG
GAGTGGGTTGCAAAAGAAGTAGGTAGCTTAACCTTCGGGAGGGCGCTTACCACTTTGTG
ATTCATGACTGGGGTGAAGTCGTAACAAGGTAACCGTA

>NR_042349.1 *Enterobacter ludwigii*
ATTGAACGCTGGCGGCAGGCCTAACACATGCAAGTCGAACGGTAGCACAGAGAGCTTG
CTCTCGGGTGACGAGTGGCGGACGGGTGAGTAATGTCTGGGAAACTGCCTGATGGAGG
GGGATAACTACTGGAAACGGTAGCTAATACCGCATAACGTCGCAAGACCAAAGAGGGG
GACCTTCGGGCCTCTTGCCATCAGATGTGCCCAGATGGGATTAGCTAGTAGGTGGGGTA
ACGGCTCACCTAGGCGACGATCCCTAGCTGGTCTGAGAGGATGACCAGCCACACTGGAA
CTGAGACACGGTCCAGACTCCTACGGGAGGCAGCAGTGGGGAATATTGCACAATGGGC
GCAAGCCTGATGCAGCCATGCCGCGTGTATGAAGAAGGCCTTCGGGTTGTAAAGTACTT
TCAGCGGGGAGGAAGGTGTTGTGGTTAATAACCGCAGCAATTGACGTTACCCGCAGAA
GAAGCACCGGCTAACTCCGTGCCAGCAGCCGCGGTAATACGGAGGGTGCAAGCGTTAA
TCGGAATTACTGGGCGTAAAGCGCACGCAGGCGGTCTGTCAAGTCGGATGTGAAATCCC
CGGGCTCAACCTGGGAACTGCATTCGAAACTGGCAGGCTAGAGTCTTGTAGAGGGGGGT
AGAATTCCAGGTGTAGCGGTGAAATGCGTAGAGATCTGGAGGAATACCGGTGGCGAAG
GCGGCCCCCTGGACAAAGACTGACGCTCAGGTGCGAAAGCGTGGGGAGCAAACAGGAT
TAGATACCCTGGTAGTCCACGCCGTAAACGATGTCGACTTGGAGGTTGTGCCCTTGAGG
CGTGGCTTCCGGAGCTAACGCGTTAAGTCGACCGCCTGGGGAGTACGGCCGCAAGGTTA
AAACTCAAATGAATTGACGGGGGCCCGCACAAGCGGTGGAGCATGTGGTTTAATTCGAT
GCAACGCGAAGAACCTTACCTACTCTTGACATCCAGAGAACTTAGCAGAGATGCTTTGG
TGCCTTCGGGAACTCTGAGACAGGTGCTGCATGGCTGTCGTCAGCTCGTGTTGTGAAAT
GTTGGGTTAAGTCCCGCAACGAGCGCAACCCTTATCCTTTGTTGCCAGCGGTCCGGCCG
GGAACTCAAAGGAGACTGCCAGTGATAAACTGGAGGAAGGTGGGGATGACGTCAAGTC
ATCATGGCCCTTACGAGTAGGGCTACACACGTGCTACAATGGCGCATACAAAGAGAAG
CGAACTCGCGAGAGCAAGCGGACCTCATAAAGTGCGTCGTAGTCCGGATTGGAGTCTGC
AACTCGACTCCATGAAGTCGGAATCGCTAGTAATCGTAGATCAGAATGCTACGGTGAAT
ACGTTCCCGGGCCTTGTACACACCGCCCGTCACACCATGGGAGTGGGTTGCAAAAGAAG
TAGGTAGCTTAACCTTCGGGAGGGCGCTTACCACTTTGTGATTCATGACTGGGGTGAAG
TCGTAACAAGGTAACCGTAGGGGAACCTGCGGCTGGATCACCTCCTTA

>NR_115005.1 *Pseudomonas oryzihabitans*
CCTAACACATGCAAGTCGAGCGGATGAGAGGAGCTTGCTCTTCGATTCAGCGGCGGACG
GGTGAGTAATGCCTAGGAATCTGCCTAGTAGTGGGGGACAACGTTTCGAAAGGAACGCT
AATACCGCATACGTCCTACGGGAGAAAGTGGGGGATCTTCGGACCTCACGCTATTAGAT
GAGCCTAGGTCGGATTAGCTAGTTGGTAGGGTAAAGGCCTACCAAGGCGACGATCCGTA
ACTGGTCTGAGAGGATGATCAGTCACACTGGAACTGAGACACGGTCCAGACTCCTACGG
GAGGCAGCAGTGGGGAATATTGGACAATGGGCGAAAGCCTGATCCAGCCATGCCGCGT
GTGTGAAGAAGGCCTTCGGGTCGTAAAGCACTTTAAGTTGGGAGGAAGGGCTCATAGC
GAATACCTGTGAGTTTTGACGTTACCAACAGAATAAGCACCGGCTAACTTCGTGCCAGC
AGCCGCGGTAATACGAAGGGTGCAAGCGTTAATCGGAATTACTGGGCGTAAAGCGCGC
GTAGGTGGCTTGATAAGTTGGATGTGAAATCCCCGGGCTCAACCTGGGAACTGCATCCA
AAACTGTCTGGCTAGAGTGCGGTAGAGGGTAGTGGAATTTCCAGTGTAGCGGTGAAATG

CGTAGATATTGGAAGGAACACCAGTGGCGAAGGCGACTACCTGGACTGACACTGACAC
TGAGGTGCGAAAGCGTGGGGAGCAAACAGGATTAGATACCCTGGTAGTCCACGCCGTA
AACGATGTCAACTAGCCGTTGGGATCCTTGAGATCTTAGTGGCGCAGCTAACGCATTAA
GTTGACCGCCTGGGGAGTACGGCCGCAAGGTTAAAACTCAAATGAATTGACGGGGGCC
CGCACAAGCGGTGGAGCATGTGGTTTAATTCGAAGCAACGCGAAGAACCTTACCTGGCC
TTGACATGCTGAGAACTTTCCAGAGATGGATTGGTGCCTTCGGGAACTCAGACACAGGT
GCTGCATGGCTGTCGTCAGCTCGTGTCGTGAGATGTTGGGTTAAGTCCCGTAACGAGCG
CAACCCTTGTCCTTAGTTACCAGCACGTTATGGTGGGCACTCTAAGGAGACTGCCGGTG
ACAAACCGGAGGAAGGTGGGGATGACGTCAAGTCATCATGGCCCTTACGGCCAGGGCT
ACACACGTGCTACAATGGTCGGTACAAAGGGTTGCCAAGCCGCGAGGTGGAGCTAATC
CCATAAAACCGATCGTAGTCCGGATCGCAGTCTGCAACTCGACTGCGTGAAGTCGGAAT
CGCTAGTAATCGTGAATCAGAACGTCACGGTGAATACGTTCCCGGGCCTTGTACACACC
GCCCGTCACACCATGGGAGTGGGTTGCTCCAGAAGTAGCTAGTCTAACCCTCGGGAGGA
CGGTTACCACGGAGTGATTCATGACTGGGGTGAAGTCGTAACAAGGTAGCCGTAGGGG

>NR_024662.1 *Pseudomonas plecoglossicida*
AGAGTTTGATCATGGCTCAGATTGAACGCTGGCGGCAGGCCTAACACATGCAAGTCGAG
CGGATGACGGGAGCTTGCTCCTTGATTCAGCGGCGGACGGGTGAGTAATGCCTAGGAAT
CTGCCTGGTAGTGGGGGACAACGTTTCGAAAGGAACGCTAATACCGCATACGTCCTACG
GGAGAAAGCAGGGGACCTTCGGGCCTTGCGCTATCAGATGAGCCTAGGTCGGATTAGCT
AGTTGGTGGGGTAATGGCTCACCAAGGCGACGATCCGTAACTGGTCTGAGAGGATGATC
AGTCACACTGGAACTGAGACACGGTCCAGACTCCTACGGGAGGCAGCAGTGGGGAATA
TTGGACAATGGGCGAAAGCCTGATCCAGCCATGCCGCGTGTGTGAAGAAGGTCTTCGGA
TTGTAAAGCACTTTAAGTTGGGAGGAAGGGCAGTAAGCTAATACCTTGCTGTTTTGACG
TTACCGACAGAATAAGCACCGGCTAACTCTGTGCCAGCAGCCGCGGTAATACAGAGGGT
GCAAGCGTTAATCGGAATTACTGGGCGTAAAGCGCGCGTAGGTGGTTCGTTAAGTTGGA
TGTGAAAGCCCCGGGCTCAACCTGGGAACTGCATCCAAAACTGGCGAGCTAGAGTACG
GTAGAGGGTGGTGGAATTTCCTGTGTAGCGGTGAAATGCGTAGATATAGGAAGGAACA
CCAGTGGCGAAGGCGACCACCTGGACTGATACTGACACTGAGGTGCGAAAGCGTGGGG
AGCAAACAGGATTAGATACCCTGGTAGTCCACGCCGTAAACGATGTCAACTAGCCGTTG
GAATCCTTGAGATTTTAGTGGCGCAGCTAACGCATTAAGTTGACCGCCTGGGGAGTACG
GCCGCAAGGTTAAAACTCAAATGAATTGACGGGGGCCCGCACAAGCGGTGGAGCATGT
GGTTTAATTCGAAGCAACGCGAAGAACCTTACCAGGCCTTGACATGCAGAGAACTTTCC
AGAGATGGATTGGTGCCTTCGGGAACTCTGACACAGGTGCTGCATGGCTGTCGTCAGCT
CGTGTCGTGAGATGTTGGGTTAAGTCCCGTAACGAGCGCAACCCTTGTCCTTAGTTACCA
GCACGTTATGGTGGGCACTCTAAGGAGACTGCCGGTGACAAACCGGAGGAAGGTGGGG
ATGACGTCAAGTCATCATGGCCCTTACGGCCTGGGCTACACACGTGCTACAATGGTCGG
TACAGAGGGTTGCCAAGCCGCGAGGTGGAGCTAATCTCACAAAACCGATCGTAGTCCG
GATCGCAGTCTGCAACTCGACTGCGTGAAGTCGGAATCGCTAGTAATCGCGAATCAGAA
TGTCGCGGTGAATACGTTCCCGGGCCTTGTACACACCGCCCGTCACACCATGGGAGTGG
GTTGCACCAGAAGTAGCTAGTCTAACCTTCGGGAGGACGGTTACCACGGTGTGATTCAT
GACTGGGGTGAAGTCGTAACAAGGTAACC

>NR_026045.1 *Pantoea ananatis*
AACACATGCAAGTCGGACGGTAGCACAGAGAGCTTGCTCTCGGGTGACGAGTGGCGGA
CGGGTGAGTAATGTCTGGGGATCTGCCCGATAGAGGGGGATAACCACTGGAAACGGTG
GCTAATACCGCATAACGTCGCAAGACCAAAGAGGGGGACCTTCGGGCCTCTCACTATCG
GATGAACCCAGATGGGATTAGCTAGTAGGCGGGGTAACGGCCCACCTAGGCGACGATC
CCTAGCTGGTCTGAGAGGATGACCAGCCACACTGGAACTGAGACACGGTCCAGACTCCT
ACGGGAGGCAGCAGTGGGGAATATTGCACAATGGGCGCAAGCCTGATGCAGCCATGCC
GCGTGTATGAAGAAGGCCTTCGGGTTGTAAAGTACTTTCAGCGGGGAGGAAGGCGATGT
GGTTAATAACCGCATTGATTGACGTTACCCGCAGAAGAAGCACCGGCTAACTCCGTGCC
AGCAGCCGCGGTAATACGGAGGGTGCAAGCGTTAATCGGAATTACTGGGCGTAAAGCG
CACGCAGGCGGTCTGTTAAGTCAGATGTGAAATCCCCGGGCTTAACCTGGGAACTGCAT
TTGAAACTGGCAGGCTTGAGTCTCGTAGAGGGGGGTAGAATTCCAGGTGTAGCGGTGAA
ATGCGTAGAGATCTGGAGGAATACCGGTGGCGAAGGCGGCCCCCTGGACGAAGACTGA
CGCTCAGGTGCGAAAGCGTGGGGAGCAAACAGGATTAGATACCCTGGTAGTCCACGCC
GTAAACGATGTCGACTTGGAGGTTGTTCCCTTGAGGAGTGGCTTCCGGAGCTAACGCGT
TAAGTCGACCGCCTGGGGAGTACGGCCGCAAGGTTAAAACTCAAATGAATTGACGGGG
GCCCGCACAAGCGGTGGAGCATATGGTTTAATTCGATGCAACGCGAAGAACCTTACCTA
CTCTTGACATCCAGAGAACTTAGCAGAGATGCTTTGGTGCCTTCGGGAACTCTGAGACA
GGTGCTGCATGGCTGTCGTCAGCTCGTGTTGTGAAATGTTGGGTTAAGTCCCGCAACGA
GCGCAACCCTTATCCTTTGTTGCCAGCGATTCGGTCGGGAACTCAAAGGAGACTGCCGG
TGATAAACCGGAGGAAGGTGGGGATGACGTCAAGTCATCATGGCCCTTACGAGTAGGG
CTACACACGTGCTACAATGGCGCATACAAAGAGAAGCGACCTCGCGAGAGCAAGCGGA
CCTCATAAAGTGCGTCGTAGTCCGGATCGGAGTCTGCAACTCGACTCCGTGAAGTCGGA
ATCGCTAGTAATCGTGGATCAGAATGCCACGGTGAATACGTTCCCGGGCCTTGTACACA
CCGCCCGTCACACCATGGGAGTGGGTTGCAAAAGAAGTAGGTAGCTTAACCTTCGGGAG
GGCGCTTACCACTTTGTGATTCATGACTGGGGTGAAGTCGTAACAAGGT

>NR_152015.1 *Achromobacter kerstersii*
ATTGAACGCTAGCGGGATGCCTTACACATGCAAGTCGAACGGCAGCACGGACTTCGGTC
TGGTGGCGAGTGGCGAACGGGTGAGTAATGTATCGGAACGTGCCTAGTAGCGGGGGAT
AACTACGCGAAAGCGTAGCTAATACCGCATACGCCCTACGGGGGAAAGCAGGGGATCG
CAAGACCTTGCACTATTAGAGCGGCCGATATCGGATTAGCTAGTTGGTGGGGTAACGGC
TCACCAAGGCGACGATCCGTAGCTGGTTTGAGAGGACGACCAGCCACACTGGGACTGA
GACACGGCCCAGACTCCTACGGGAGGCAGCAGTGGGGAATTTTGGACAATGGGGGAAA
CCCTGATCCAGCCATCCCGCGTGTGCGATGAAGGCCTTCGGGTTGTAAAGCACTTTTGG
CAGGAAAGAAACGTCATGGGCTAATACCCCGTGAAACTGACGGTACCTGCAGAATAAG
CACCGGCTAACTACGTGCCAGCAGCCGCGGTAATACGTAGGGTGCAAGCGTTAATCGGA
ATTACTGGGCGTAAAGCGTGCGCAGGCGGTTCGGAAAGAAAGATGTGAAATCCCAGAG
CTTAACTTTGGAACTGCATTTTTAACTACCGAGCTAGAGTGTGTCAGAGGGAGGTGGAA
TTCCGCGTGTAGCAGTGAAATGCGTAGATATGCGGAGGAACACCGATGGCGAAGGCAG
CCTCCTGGGATAACACTGACGCTCATGCACGAAAGCGTGGGGAGCAAACAGGATTAGA
TACCCTGGTAGTCCACGCCCTAAACGATGTCAACTAGCTGTTGGGGCCTTCGGGCCTTG
GTAGCGCAGCTAACGCGTGAAGTTGACCGCCTGGGGAGTACGGTCGCAAGATTAAAAC
TCAAAGGAATTGACGGGGACCCGCACAAGCGGTGGATGATGTGGATTAATTCGATGCA

ACGCGAAAAACCTTACCTACCCTTGACATGTCTGGAATTCCGAAGAGATTYGGAAGTGC
TCGCAAGAGAACCGGAACACAGGTGCTGCATGGCTGTCGTCAGCTCGTGTCGTGAGATG
TTGGGTTAAGTCCCGCAACGAGCGCAACCCTTGTCATTAGTTGCTACGAAAGGGCACTC
TAATGAGACTGCCGGTGACAAACCGGAGGAAGGTGGGGATGACGTCAAGTCCTCATGG
CCCTTATGGGTAGGGCTTCACACGTCATACAATGGTCGGGACAGAGGGTCGCCAACCCG
CGAGGGGGAGCCAATCCCAGAAACCCGATCGTAGTCCGGATCGCAGTCTGCAACTCGA
CTGCGTGAAGTCGGAATCGCTAGTAATCGCGGATCAGCATGTCGCGGTGAATACGTTCC
CGGGTCTTGTACACACCGCCCGTCACACCATGGGAGTGGGTTTTACCAGAAGTAGTTAG
CCTAACCGCAAGGGGGGCGATTACCACGGTAGGATTCATGACTGGGGTGAAGTCGTAA
CAAGGTAGCCGTATCGGAAGG

>NR_074936.1 *Xanthomonas campestris*
TAAGTGAAGAGTTTGATCCTGGCTCAGAGTGAACGCTGGCGGCAGGCCTAACACATGCA
AGTCGAACGGCAGCACAGTAAGAGCTTGCTCTTATGGGTGGCGAGTGGCGGACGGGTG
AGGAATACATCGGAATCTACTCTTTCGTGGGGGATAACGTAGGGAAACTTACGCTAATA
CCGCATACGACCTACGGGTGAAAGCGGAGGACCTTCGGGCTTCGCGCGATTGAATGAGC
CGATGTCGGATTAGCTAGTTGGCGGGGTAAAGGCCCACCAAGGCGACGATCCGTAGCTG
GTCTGAGAGGATGATCAGCCACACTGGAACTGAGACACGGTCCAGACTCCTACGGGAG
GCAGCAGTGGGGAATATTGGACAATGGGCGCAAGCCTGATCCAGCCATGCCGCGTGGG
TGAAGAAGGCCTTCGGGTTGTAAAGCCCTTTTGTTGGGAAAGAAAAGCAGTCGGTTAAT
ACCCGATTGTTCTGACGGTACCCAAAGAATAAGCACCGGCTAACTTCGTGCCAGCAGCC
GCGGTAATACGAAGGGTGCAAGCGTTACTCGGAATTACTGGGCGTAAAGCGTGCGTAG
GTGGTGGTTTAAGTCTGTTGTGAAAGCCCTGGGCTCAACCTGGGAATTGCAGTGGATAC
TGGGTCACTAGAGTGTGGTAGAGGGTAGCGGAATTCCCGGTGTAGCAGTGAAATGCGTA
GAGATCGGGAGGAACATCCGTGGCGAAGGCGGCTACCTGGACCAACACTGACACTGAG
GCACGAAAGCGTGGGGAGCAAACAGGATTAGATACCCTGGTAGTCCACGCCCTAAACG
ATGCGAACTGGATGTTGGGTGCAATTTGGCACGCAGTATCGAAGCTAACGCGTTAAGTT
CGCCGCCTGGGGAGTACGGTCGCAAGACTGAAACTCAAAGGAATTGACGGGGGCCCGC
ACAAGCGGTGGAGTATGTGGTTTAATTCGATGCAACGCGAAGAACCTTACCTGGTCTTG
ACATCCACGGAACTTTCCAGAGATGGATTGGTGCCTTCGGGAACCGTGAGACAGGTGCT
GCATGGCTGTCGTCAGCTCGTGTCGTGAGATGTTGGGTTAAGTCCCGCAACGAGCGCAA
CCCTTGTCCTTAGTTGCCAGCACGTAATGGTGGGAACTCTAAGGAGACCGCCGGTGACA
AACCGGAGGAAGGTGGGGATGACGTCAAGTCATCATGGCCCTTACGACCAGGGCTACA
CACGTACTACAATGGTAGGGACAGAGGGCTGCAAACCCGCGAGGGTAAGCCAATCCCA
GAAACCCTATCTCAGTCCGGATTGGAGTCTGCAACTCGACTCCATGAAGTCGGAATCGC
TAGTAATCGCAGATCAGCATTGCTGCGGTGAATACGTTCCCGGGCCTTGTACACACCGC
CCGTCACACCATGGGAGTTTGTTGCACCAGAAGCAGGTAGCTTAACCTTCGGGAGGGCG
CTTGCCACGGTGTGGCCGATGACTGGGGTGAAGTCGTAACAAGGTAGCCGTATCGGAAG
GTGCGGCTGGATCACCTCCTTT

>NR_075056.2 *Aquifex aeolicus*
TCCCTGAAGAGTTTGATCCTGGCTCAGCGCGAACGCTGGCGGCGTGCCTAACACATGCA
AGTCGTGCGCAGGGTCGGCCCCTTTTGGGGCCGGCCGCTGAGCGGCAAACGGGTGAGTA
AC

ACGTGGGTAACCTACCCCCAGGAGGGGGATAACCCCGGGAAACCGGGGCTAATACCCC
ATAATGCCACCCGCCACTAAGGCGCGGTGGCCAAAGGGGGCCTCTGGCCTTCGGCCACG
CTCCCGCCTGGGGATGGGCCCGCGGCCCATCAGGTAGTTGGTGGGGTAACGGCCCACCA
AGCCTATGACGGGTAGCCGGCCTGAGAGGGTGGCCGGCCACAGCGGGACTGAGACACG
GCCCGCACCCCTACGGGGGGCAGCAGTGGGGAATCGTGGGCAATGGGCGAAAGCCTGA
CCCCGCGACGCCGCGTGGGGGATGAAGCCCTGCGGGGTGTAAACCCCTGTCGGGGGGG
ACGATGCGGACACGGGTTAATAGCCCGTGTCCGTGACGGTACCCCCAGAGGAAGGGAC
GGCTAACTACGTGCCAGCAGCCGCGGTAATACGTAGGTCCCAAGCGTTGCGCGAAGTCA
CTGGGCGTAAAGCGTCCGCAGCCGGTCGGGTAAGCGGGATGTCAAAGCCCACGGCTCA
ACCGTGGACCGGCATCCCGAACTGCCCGACTTGAGGCACGCCCGGGCAGGCGGAATTCC
CGGGGTAGCGGTGAAATGCGTAGATCTCGGGAGGAACACCGAAGGGGAAGCCAGCCTG
CTGGGGCTGTCCTGACGGTCAGGGACGAAAGCCGGGGGAGCAAACCGGATTAGATACC
CGGGTAGTCCCGGCCGTAAACCATGGGCGCTAGGGCTTGTCCCTCTGGGGCAGGCTCGC
AGCTAACGCGTTAAGCGCCCCGCCTGGGGAGTACGGGCGCAAGCCTGAAACTCAAAGG
AATTGGCGGGGGCCCGCACAACCGGTGGAGCGTCTGGTTCAATTCGATGCTAACCGAAG
AACCTTACCCGGGCTTGACATGCCGGGGAGACTCCGCGAAAGCGGAGTTGTGGAAGTCC
CTAGGACTTTCCCCCGGCACAGGTGGTGCATGGCCGTCGTCAGCTCGTGTCGTGAGATG
TTGGGTTAAGTCCCGCAACGAGCGCAACCCCTGCCCCCAGTTGCTACCCCGTTTGGGGA
GCACTCTGGGGGGACCGCCGGCGATAAGCCGGAGGAAGGGGGGGATGACGTCAGGTCA
GTATGCCCTTTATGCCCGGGGCCACACAGGCGCTACAGTGGCCGGGACAATGGGATGCG
ACCCCGTAAGGGGGAGCTAATCCCTAAACCCGGTCATGGTGCGGATTGGGGGCTGAAA
CTCGCCCCCATGAAGCCGGAATCGGTAGTAACGGGGTATCAGCGATGTCCCCGTGAATA
CGTTCTCGGGCCTTGCACACACCGCCCGTCACGCCACGGAAGTCGGTCCGGCCGGAAGT
CCCCGAGCTAACCGGCTCCTTCGGGAGCCGGAGGCAGGGGCCGATGGCCGGGCCGGCG
ACTGGGGCGAAGTCGTAACAAGGTAGCCGTAGGGGAACCTGCGGCTGGATCACCTCCTT

AIMS Press

© 2021 the Author(s), licensee AIMS Press. This is an open access article distributed under the terms of the Creative Commons Attribution License (http://creativecommons.org/licenses/by/4.0)
